# Structural studies and molecular dynamics simulations suggest a processive mechanism of exolytic lytic transglycosylase from *Campylobacter jejuni*

**DOI:** 10.1371/journal.pone.0197136

**Published:** 2018-05-14

**Authors:** Jagamya Vijayaraghavan, Vijay Kumar, Nikhil P. Krishnan, Ross T. Kaufhold, Ximin Zeng, Jun Lin, Focco van den Akker

**Affiliations:** 1 Department of Biochemistry, Case Western Reserve University, Cleveland, OH, United States of America; 2 Institute of agriculture, University of Tennessee, Knoxville, TN, United States of America; Friedrich-Alexander-Universitat Erlangen-Nurnberg, GERMANY

## Abstract

The bacterial soluble lytic transglycosylase (LT) breaks down the peptidoglycan (PG) layer during processes such as cell division. We present here crystal structures of the soluble LT Cj0843 from *Campylobacter jejuni* with and without bulgecin A inhibitor in the active site. Cj0843 has a doughnut shape similar but not identical to that of *E*. *coli* SLT70. The C-terminal catalytic domain is preceded by an L-domain, a large helical U-domain, a flexible linker, and a small N-terminal NU-domain. The flexible linker allows the NU-domain to reach over and complete the circular shape, using residues conserved in the Epsilonproteobacteria LT family. The inner surface of the Cj0843 doughnut is mostly positively charged including a pocket that has 8 Arg/Lys residues. Molecular dynamics simulations with PG strands revealed a potential functional role for this pocket in anchoring the negatively charged terminal tetrapeptide of the PG during several steps in the reaction including homing and aligning the PG strand for exolytic cleavage, and subsequent ratcheting of the PG strand to enhance processivity in degrading PG strands.

## Introduction

The bacterial cell wall of Gram-negative bacteria contains a peptidoglycan (PG) sacculus between the outer and inner membranes. The PG is critical to maintain structural and physical integrity of bacteria. The PG layer is a dynamic structure as it needs to be broken down and remodeled during cell growth [1], cell division [2, 3], or during formation of large periplasm-spanning structures like flagella or pili [4, 5]. The PG layer is composed of saccharide and amino acid-type building blocks with the saccharide component being a polymer of *N*-acetylglucosamine (GlcNAc) and *N*-acetylmuramic acid (MurNAc). The PG building block GlcNAc-MurNAc-pentapeptide (part of lipid II) is utilized by transglycosylases to polymerase the PG saccharide chains [6]. Transpeptidases can subsequently crosslink the peptide moieties of adjacent PG glycan strands. Certain penicillin binding proteins (PBP) can harbor both the transglycosylase and transpeptidase activity. The breakdown of the PG involves a number of enzymes including carboxypeptidases, endopeptidases, amidases, and lytic transglycosylases (LT). The LT enzymes cleave the PG MurNAc-GlcNAc glycosidic bond similar to lysozyme [7] with the noted difference that LTs have an additional (2^nd^) cyclization step; this step requires a boat conformation of MurNAc leading to a 1,6-anhydromuramyl product as the predominant terminal saccharide moiety [8, 9]. The active sites of both lysozyme and LT have a conserved glutamic acid which is proposed to act as a proton donor during catalysis of PG degradation [10–12]. *E*. *coli* deficient in LT do not exhibit defects in cell growth [13] yet such LT mutants can be hypersensitive to antibiotics that inhibit cell wall synthesis [14]. Furthermore, a known inhibitor of LT activity, bulgecin A, is known to synergistically inhibit bacterial growth in the presence of β-lactam antibiotics in several Gram-negative bacteria [15, 16]. Therefore, LTs provide an attractive target for the design of inhibitors that can be used in combination with β–lactams to counteract bacterial infections.

Bacterial LTs can be membrane bound or soluble; they are divided into different families and several subfamilies based on their conserved motifs [10, 17, 18]. Although the overall sequence identity between different LTs is relatively limited, the catalytic glutamic acid and catalytic domain fold are strongly conserved in different families and organisms. We report here structure-function studies of soluble periplasmic LT of *Campylobacter jejuni*. The LT Cj0843 was identified from a virulent strain of *C*. *jejuni* and localized in the periplasm [19]. Also, Cj0843 was found to play a role in β–lactam resistance [19], and its expression to increase by the presence of mucins [20]. Moreover, Cj0843 mutants were also defective in cecal colonization in chickens [19]. These results suggest that inhibition of Cj0843 could potentially be therapeutically beneficial to treat campylobacteriosis, one of the most common human infections. Here, we present the crystal structures of LT Cj0843 in the absence and presence of the inhibitor bulgecin A. Furthermore, we carried out biophysical and biochemical analyses as well as molecular dynamics (MD) simulations with PG fragments to yield insights into PG degradation by doughnut-shaped LTs.

## Results

### Overall structure of *C*. *jejun*i Cj0843

**Fig 1:**
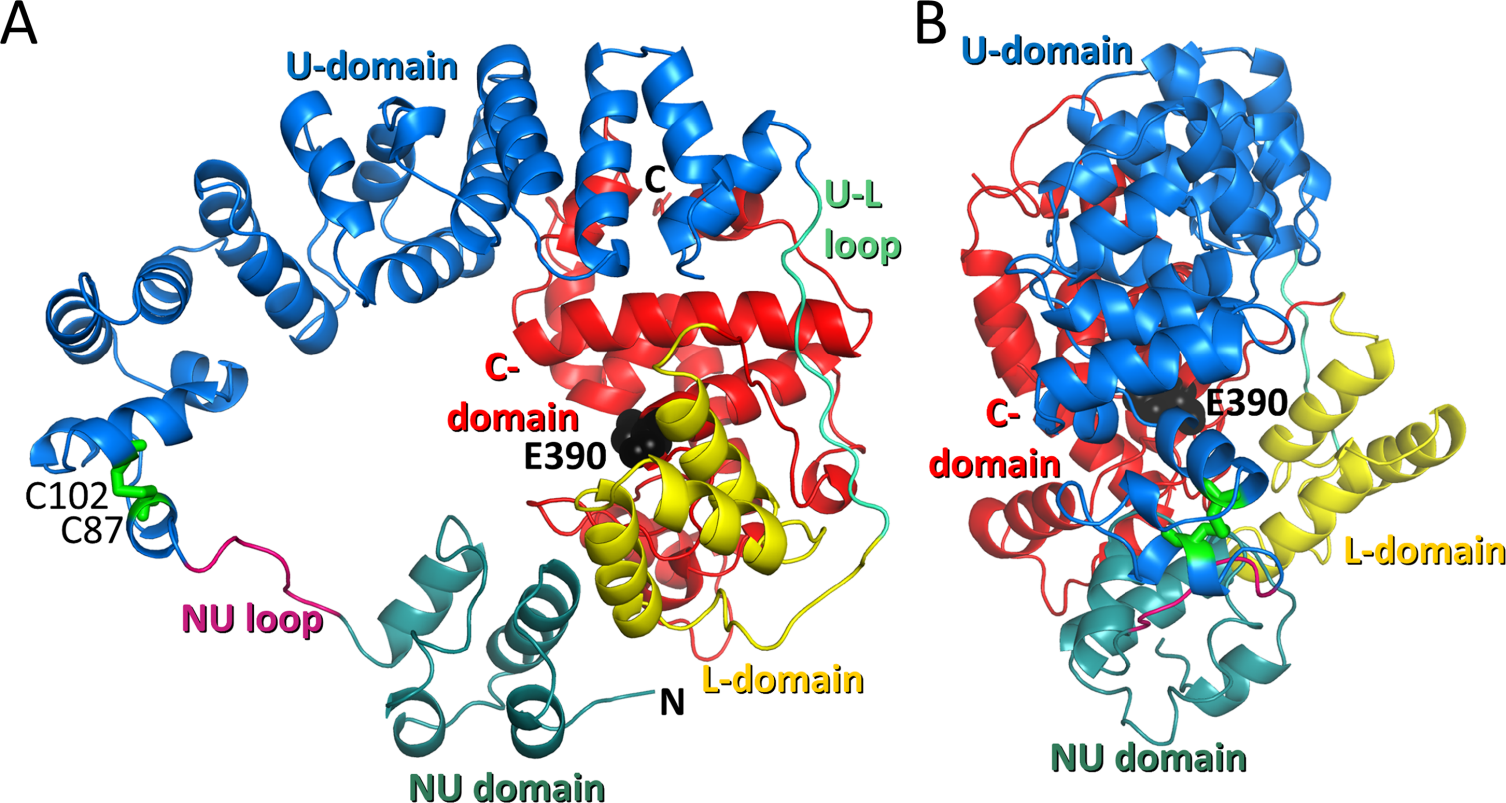
The structure of soluble lytic transglycosylase of Cj0843. A Front view of Cj0843 depicting the NU domain (teal), NU-loop (magenta), U-domain (blue), UL-loop (blue-green), L domain (yellow), and C-domain (red). The disulfide bond between C87 and C102 is in green stick model, and the catalytic E390 is shown in black spheres. B Side view of Cj0843 (90 degrees rotated along a vertical axis relative to the orientation in A).

Both Cj0843 crystal forms reveal an overall protein structure, at 1.87 and 2.28 Å resolution, that adopts a doughnut shape formed by four domains (Fig 1). The structures from both crystal forms are very similar with a root-mean-square-deviation (RMSD) for 509 Cα atoms of 0.75 Å. At the N-terminus is a small helical domain (NU-domain) connected *via* a flexible linker to the helical U-domain. This flexible linker (residues 75–83) is partially resolved in the P3_1_21 crystal form and fully resolved in the I23 crystal form; it spans 22Å between ordered anchor points I74 and Y84 in the NU-domain and U-domain, respectively. The U-domain anchor point is part of helix α5; this helix is tethered to helix α7 of the U-domain *via* a disulfide bond between C87 and C102. The other end of the U-domain is connected to a distant L-domain *via* a long ordered UL-loop (Fig 1). Finally, the L-domain is connected to the C-terminal catalytic C-domain. The L- and C-domains of the protein complete the circular doughnut shape as both interact with the NU-domain. The central pore of the ‘doughnut’ is elliptical and has an inner diameter ranging from 16 to 37Å.

*C*. *jejuni* is unusual in that it can glycosylate proteins; native Cj0843 was observed to have at least 5 N-linked glycosylation sites, 4 of which had been mapped by mass spectrometry [21]. These glycosylated residues are in the U-domain (N99 and N175), L-domain (N329), and C-domain (N376). All four sites are located on the side face or outer perimeter of the doughnut-shaped Cj0843 and are thus distant from the active site (S1 Fig; note that the *E*. *coli* expressed Cj0843 is not glycosylated). The exact functional importance of this glycosylation is not known yet general protein glycosylation in *C*. *jejuni* likely increases bacterial viability [22].

**Fig 2:**
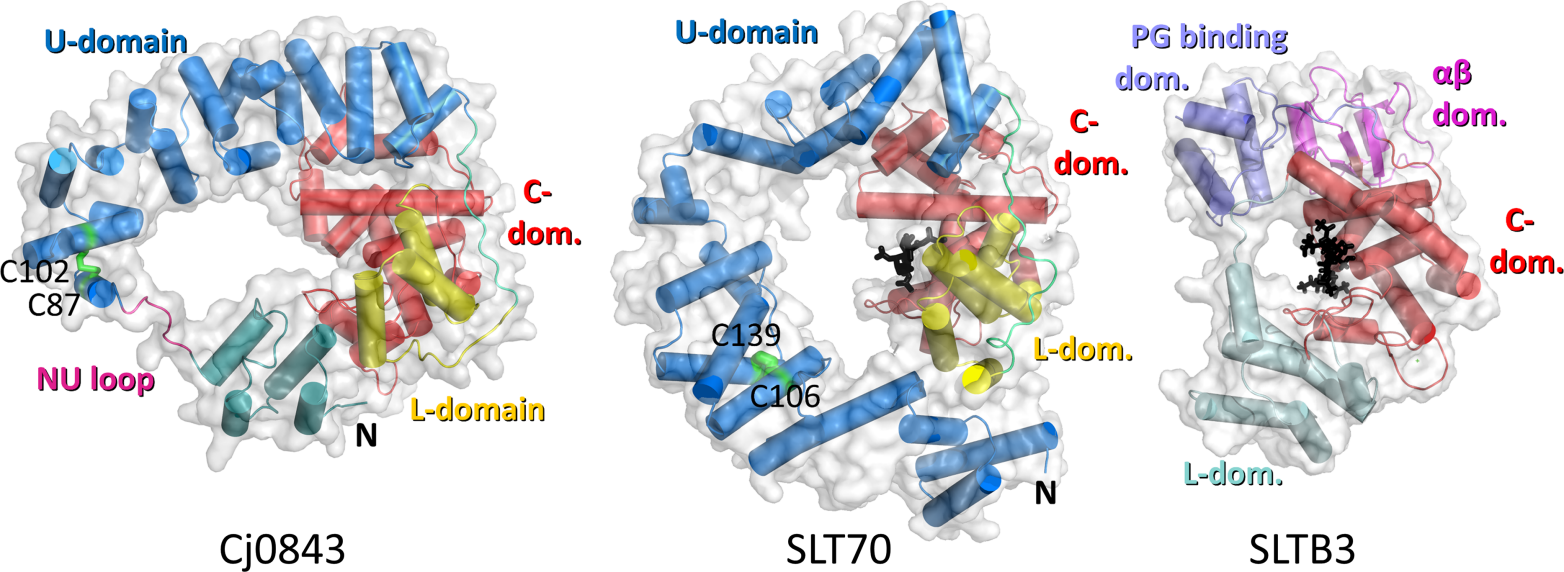
Structure comparison of Cj0843, SLT70, and SLTB3. The proteins are depicted with a transparent surface and cartoon representation. Cj0843 is shown with the same coloring scheme as in Fig 1. *E*. *coli* SLT70 (PDB ID: 1QTE; [24]) has a similar coloring scheme except it does not have an NU-domain. The SLT70 structure includes a 1,6-anhydromurotripeptide (black sticks) to highlight the location of the active site; the disulfide bond is shown in green. The *P*. *aeruginosa* SLTB3 (PDB ID: 5A07)[27] is depicted with a similar muropeptide ligand shown in black sticks; the N-terminal domain (light blue), catalytic domain (red), PG binding domain (purple), and αβ-domain (pink) are shown.

A structural similarity search of Cj0843 using DALI [23] found the *E*. *coli* soluble lytic transglycosylase SLT70 [8, 24, 25] to have the highest structurally similarity yielding a Z-score of 22.3, representing the number of standard deviations above the mean for this similarity score, and RMSD of 4.5Å for 371 aligned residues with a sequence identity of 19% (PDB ID 1SLY; Fig 2 and S2 Fig). This structural similarity is remarkable with the most substantial sequence similarity present in the catalytic C-domain (S2 Fig). Recently, the structure of LtgA from *Neisseria meningitides* was determined and found to be very similar to SLT70 [26]. Like Cj0843, SLT70 is predominantly an α-helical protein yet about 100 residues longer (645 compared to 541 amino acids for Cj0843; their predicted signal peptide lengths are 27 and 19 amino acids, resp.). Other structurally similar proteins are several membrane LTs such as MltC (PDB ID 4CFP), MltE (PDB ID 2Y8P), MltF (PDB ID 4OYV) with Z-scores around 12–13 (for about 150 aligned residues mostly comprising part of the catalytic domain). The DALI search also found another soluble lytic transglycosylase, SLTB3 of *Pseudomonas aeruginosa* that was structurally similar to Cj0843 with a Z-score of 6.6 and RMSD of 4.9Å for 147 aligned residues (PDB ID 5ANZ)[27]. Interestingly, both *E*. *coli* and *P*. *aeruginosa* soluble LTs adopt a doughnut shape, similar to Cj0843 (Fig 2). The central holes in both Cj0843 and SLT70 are however much larger than that of SLTB3. The crystal structures of these proteins co-crystallized with muropeptides indicating the binding position of PG saccharide moieties (Fig 2). The circular structure of both SLT70 from *E*. *coli* and SLTB3 *P*. *aeruginosa* suggest that the PG can thread through the hole near the active site of the LT.

Cj0843 shares 29–34% sequence similarity with other members of the Epsilonproteobacteria family (S2 Fig) such as the exolytic Slt from *Helicobacter pylori*[28]; this organism has a postulated link to peptic ulcers and cancer [29, 30]. Their most substantial similarity is located in the catalytic C-domain (S2 Fig). The other domains share less sequence conservation in the epsilonproteobacteria LT family yet are nevertheless more similar to each other than to the gammaproteobacteria *E*. *coli* SLT70 (S2 Fig). The sequence alignment highlights several regions of sequence conservation that could point to critical functional roles and will be discussed below.

### U-domain

The U-domains of Cj0843 and SLT70 are more structurally divergent compared to the other domains. The U-domain of Cj0843 is smaller than SLT70 as it has only 20 α-helices (including the NU-domain helices) compared to SLT70 which has 22 (Figs 1 and 2). Also, the α-helices in Cj0843 are in general shorter and not as regularly arranged compared to the supra-helical arrangement in SLT70. An additional difference is that Cj0843 has a split U-domain with a separate N-terminal NU-domain (comprising α1- α4). Being larger, the U-domain of SLT70 completes the circular shape as an entire rigid U-shaped unit without the NU-domain-linker configuration in Cj0843. The Cj0843 U-domain contains a disulfide bond between C87 and C102; it is noteworthy that SLT70 (and LtgA) also contains a disulfide bond in the U-domain (between C106 and C139) providing also structural stability at a similar position along its circular shape (Figs 1 and 2). The importance of the disulfide bond for stability was probed using differential scanning fluorimetry (DSF) using the SYPRO orange dye; removing the disulfide bond with reducing agents decreased the melting temperature of Cj0843 from 51.5 to 46.0, a 5.5°C destabilization (Figure A in S3 Fig).

A superposition of the Cj0843 U-domain with that in STL70 reveals that the structural similarity in this domain starts with helix α14 (helix 16 in SLT70) and ends with helix α20. The sequence conservation between these two proteins is very limited in this α14-α20 section, in particular near its N-terminus (S2 Fig). A DALI search using just the U-domain revealed a structural similarity to *Shigella flexneri* type III secretion chaperone IpgC (Z-score 8.6, RMSD of 2.6Å) despite sharing only 13% sequence identity. Seven of the helices of the U-domain of Cj0843 (α14-α20) superimpose onto the equivalent helices in the TPR motif-containing IpgC. IpgC forms a functional dimer and binds the IpaB peptide in the major curved groove [31]. The Cj0843 U-domain uses equivalent faces of its similar 7 helix section to interact with the rest of the U-domain and with the C-domain.

### L-domain

The L-domain in Cj0843 is structurally equivalent to the L-domain in SLT70 situated in the same position flanking the C-domain (Fig 2). The L-domain in Cj0843 has three long α-helices and is smaller compared to the L-domain in SLT70 L-domain which contains 6 helices (4 large and 2 small). The three α helices in Cj0843 are structurally equivalent to 3 of the 4 large helices of the SLT70 L-domain. Like Cj0843, the other members of the epsilonproteobacteria families have the smaller L-domain configuration (S2 Fig).

A DALI search with just the Cj0843 L-domain revealed structural similarity to AscG of *Aeromonas hydrophila*, a chaperone component of the type III secretion system (Z-score of 5.1, RMSD 2.6Å for 44 residues). AscG, made up of one-and-a-half TPR motif units, was crystallized in complex with another chaperone AscE [32]. AscG uses its helices for protein-protein interactions similar to the corresponding L-domain in LTs interacting with the other LT domains.

### C-domain

The C-domains or the catalytic domains of the Cj0843 and SLT70 proteins are structurally very similar in accord with their higher sequence conservation. Most of the active site residues are conserved (S2 and S4 Figs as calculated using ConSurf [33]) including the catalytic glutamic acid (E390) which is located at the end of helix α25.

**Fig 3:**
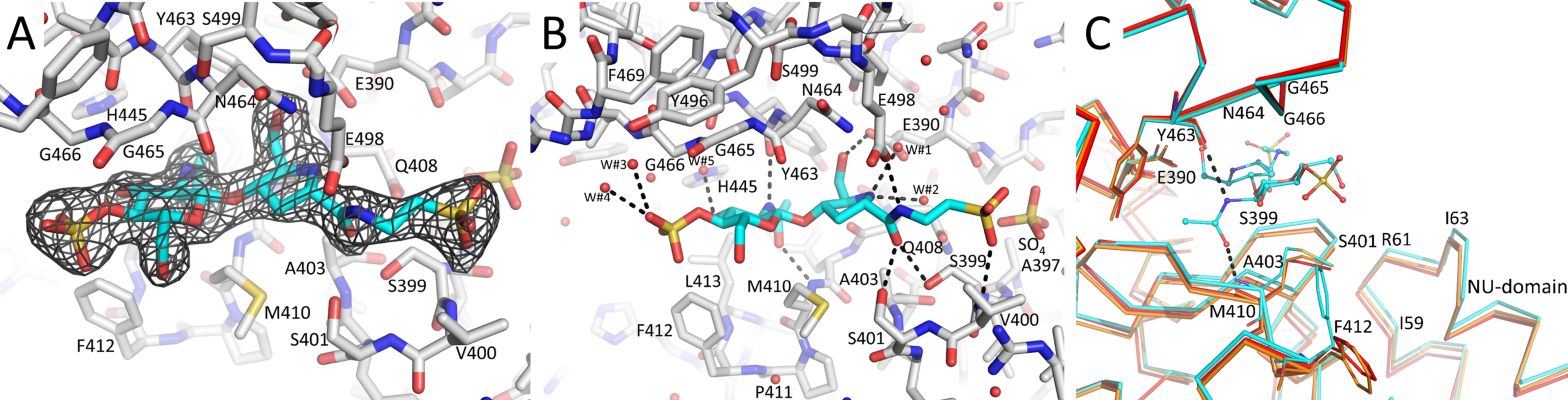
Bulgecin A binding to Cj0843. A Unbiased |Fo|-|Fc| difference density contoured at 3σ contour level showing the presence of bulgecin A in the active site (bulgecin A was removed from the map calculations). Bulgecin A is depicted with carbon atoms colored in cyan. B Interactions of bulgecin A in the active site; interacting water molecules are shown as red spheres. Hydrogen bonds are depicted as dashed lines. C Active site movements of Cj0843 upon bulgecin A binding. The bulgecin A complex (cyan), apo Cj0843 P3_1_21 structure (red), apo Cj0843 I23 structure (orange) are superimposed to highlight the main chain movements and the F412 side chain movement. The view is roughly 90° rotated from the view in A and depicts the active site groove from the side. Residues M410, Y463, and the catalytic E390 are shown in stick model.

A common active site inhibitor of LTs is the natural product bulgecin A which is known to inhibit *E*. *coli* SLT70 [8], *H*. *pylori* Slt [15], *E*. *coli* MltE [34], and *N*. *meningitidis* LtgA [26]. To probe whether bulgecin A can also bind to Cj0843, we determined the structure of bulgecin A bound to Cj0843 to 2.04 Å resolution (Fig 3). Bulgecin A binds in the active site groove with several chemical groups; one of these groups is *N*-acetyl-glucose moiety, a moiety also found in the PG substrate. The *N*-acetyl group hydrogen bonds with the backbone oxygen of Y463 and backbone nitrogen of M410 (Fig 3B). The hydroxymethyl moiety of the pyrrolidine ring forms a hydrogen bond with the catalytic E390; the pyrrolidine ring nitrogen, likely protonated and mimicking a transition state, is 3.6Å from the carboxyl group of E390 (Fig 3B). The amide moiety of the pyrrolidine ring forms additional hydrogen bonding interactions in the active site with S399, S401, and E498. The two sulfate moieties of bulgecin A interact either directly or indirectly with the protein; one of the sulfates is situated near the N-terminal end of the α29 helix (near F469) likely providing a favorable helix dipole moment interaction. Bulgecin A binds to the active site of Cj0843 and induces conformational changes that lead to narrowing of the active site groove (Fig 3C). The distance between M410 and Y463 backbone atoms that hydrogen bond with the *N*-acetyl group decrease from 8.3Å in both unbound structures to 7.5Å in the bulgecin A complex structure (Fig 3C). Additional clamping movements involve the loop 399–402 and concomitant movement of the NU-domain including residues 59–63. Also, the side chain of F412 flips towards bulgecin A to make 3.5Å anion-pi interactions [35] with one of the sulfate moieties of bulgecin A (Fig 3C). The interaction between Cj0843 and bulgecin A was probed using DSF showing that binding of bulgecin A increased the thermostability of Cj0843 by 9°C (Figure B in S3 Fig). This result indicates a strong stabilizing effect of bulgecin A on the Cj0843 structure in agreement with the observation that the active site clamps down on bulgecin A upon binding (Fig 3C). We subsequently carried out additional DSF measurements of Cj0843 with varying bulgecin A concentrations (only up to 640 μM due to limited availability of the bulgecin A compound, and DMSO concentration considerations); the measured K_d_ of bulgecin A binding to Cj0843 is 210 μM ± 28 μM (Figure C in S3 Fig).

**Fig 4:**
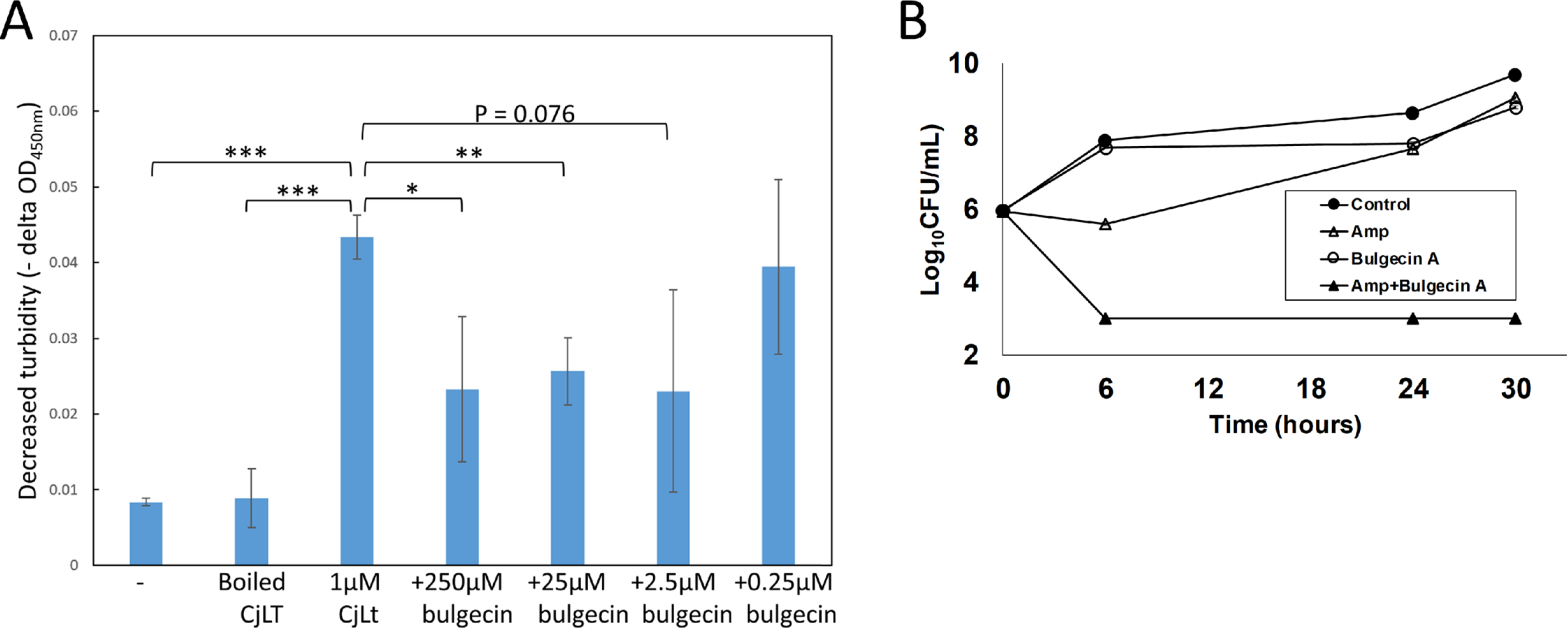
Enzymatic and microbiological assays of Cj0843 with bulgecin A. A Turbidity assay shows inhibition of 1μM Cj0843 by bulgecin A (0.25–250μM). Control experiments include adding no enzyme and boiled/inactivated Cj0843. Data show the mean ± SEM of three independent experiments. *P<0.05, **P<0.01, and ***P<0.001 (Student’s t-test). B Bulgecin A potentiated the efficacy of Amp against β-lactam resistant *C*. *jejuni*. A β-lactam resistant *C*. *jejuni* strain was inoculated in MH broth (control), or MH broth supplemented with Amp (128 μg/mL, sublethal concentration), bulgecin A (100 μg/mL) or both Amp (128 μg/mL) and bulgecin A (100 μg/mL). The detection limit (dotted line) of the assay is 1 x 10^3^ CFU/mL. Each data point represents the mean ± SEM obtained from duplicate wells in the microtiter plate growth assay. At 30hr, MH vs Amp: P = 0.01; Amp vs Bul: P = 0.04; Bul vs Amp+Bul: P = 0.0001; Amp vs Amp+Bul: P < 0.0001 (Student’s t-test).

The bulgecin A interactions are similar to those observed for other bulgecin A LT complexes including SLT70 [8], SLT35[36], MltE [34], and LtgA [26]. The interacting residues involved are also relatively conserved in the *H*. *pylori* LT, an epsilonproteobacteria LT family member known to be inhibited by bulgecin A [15](S2 Fig). In concord with the above results, we find that bulgecin A inhibits Cj0843 activity as measured *via* a turbidity assay using substrate PG from the *C*. *jejuni* LT mutant (Fig 4). Around 50% inhibition is observed at bulgecin A concentrations ranging from 2.5–250μM; a lower concentration of 0.25μM was ineffective as no significant inhibition is to be expected since the 1μM Cj0843 protein concentration in the assay was in excess (Fig 4A). Consistent with this finding, bulgecin A was also observed to increase the susceptibility of β-lactam resistant *C*. *jejuni* to Amp (Fig 4B). The Amp resistant *C*. *jejuni* strain could be rapidly killed in the presence of both Amp and bulgecin A after 6 hr of treatment despite its normal growth in the presence of Amp or bulgecin A alone (Fig 4B).

### Inter-domain linkers

Two inter-domain loops or linkers are present in Cj0843. The NU-loop is not well ordered and has an overall basic charge as it contains two lysines. Also, this NU-loop has two prolines which are a common occurrence in mobile linker regions perhaps limiting some flexibility and keeping it away from the attached domain [37]. The second linker, the UL-loop, connects the U-domain to L-domain and is well ordered, solvent accessible, and in a roughly similar position in SLT70 yet with limited sequence conservation (Figs 1 and 2 and S2 Fig). Both linker regions (L70-A85 and E289-Q313) were predicted from the amino acid sequence by the DLP-SVM domain-linker predicting server [38].

### The NU-domain and its interactions with the C-/L-domains

The NU-domain is comprised of 4 antiparallel α-helices and has limited structural homologs in the Protein Data Bank. The nearest structural neighbor is the N-terminal domain in translation initiator factor eIF2b-like protein (DALI Z-score of 4.6; RMSD of 2.1Å for 54 Cα; 9% sequence identity; PDBid 4ZEM). The small NU-domain is held together by a hydrophobic core containing I22, L25, I38, Y39, L42, L47, L55, I59, I66, and l70, as well as hydrogen bonds between the conserved D35 and backbone nitrogens of F60 and R61. The NU-domain contacts the C-domain and part of the L-domain through hydrogen bonds, salt bridges, hydrophobic, and van der Waals interactions. NU-domain residue R40 forms a salt-bridge with C-domain residue D428 (S5 Fig) whereas NU-domain residues Y36 and Y37 hydrogen bond with D430. The interface also includes van der Waals interactions involving NU-domain residues L25, E28, S31, L32, A33, Y39, R65, and I66. The complementary surface of this interface is comprised of L-domain residues P315, F316, and Q319 and C-domain residues I398, Y402, L404, and F433 (S5 Fig). The buried surface at this interface is 678Å^2^ with 57% of this surface being hydrophobic (calculated using PISA [39]). The residues of the NU-domain and the C-/L-domain interface are relatively conserved in the epsilonproteobacteria but not in SLT70 as its U-domain contacts only the L-domain (Fig 2 and S2 and S4 Figs). This linker-tethered NU-domain and its interactions with the rest of Cj0843 is one of the most salient differences between the structures of Cj0843 and SLT70, and suggests an important conserved functional requirement to maintain the circular nature of soluble LTs in the epsilonproteobacteria family.

### Conserved electrostatic surface features and implications for interactions with PG

**Fig 5:**
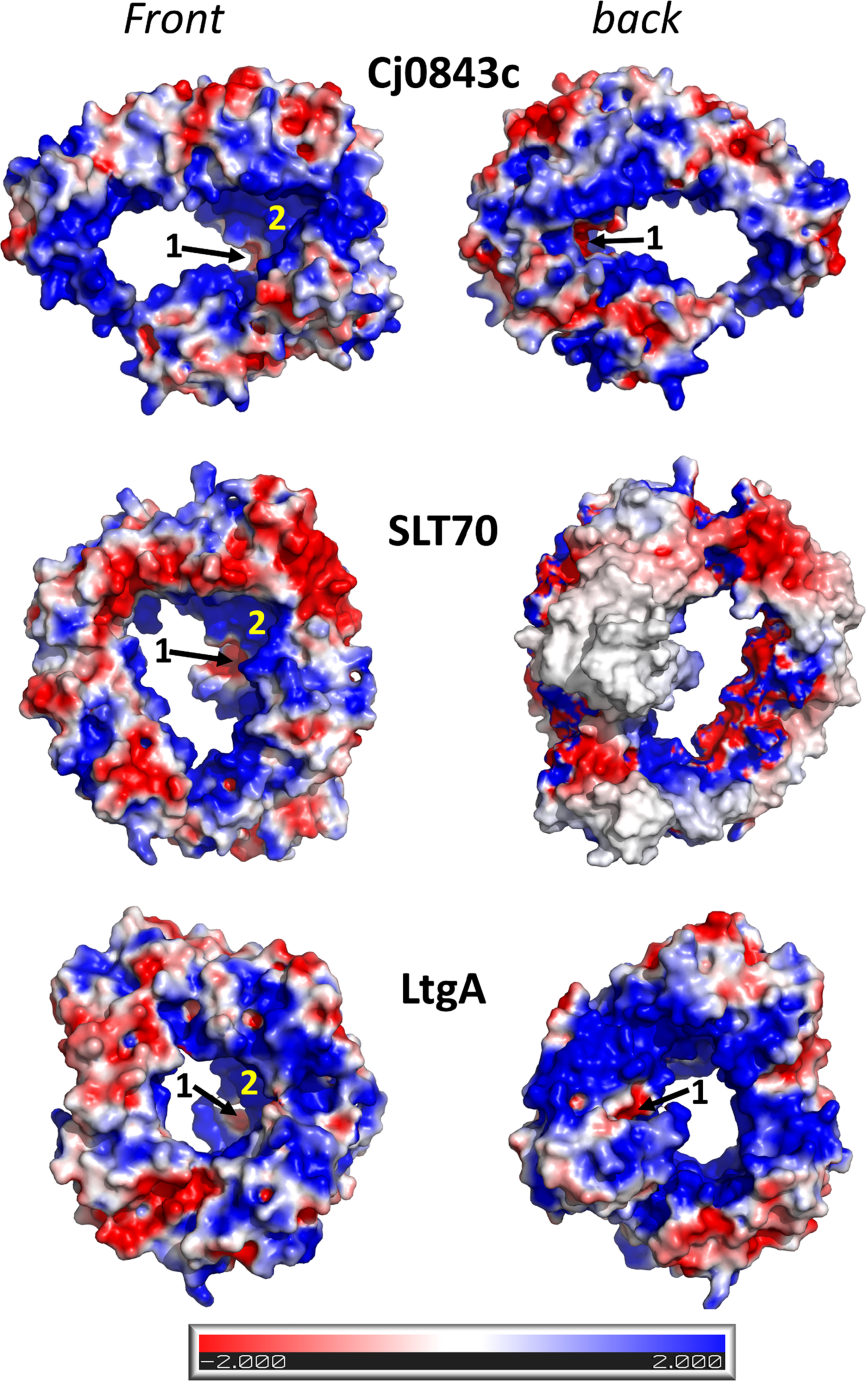
The electrostatic surface potential of Cj0843, SLT70, and LtgA. The electrostatic surface calculations were done using APBS [41], and two opposite views of the doughnut-shaped proteins are shown. The active site groove is indicated by an arrow and labeled ‘1’; the positively charged pocket 2 is indicated by a yellow ‘2’.

The most prominent feature of the electrostatic potential surface of Cj0843 is its predominantly positively charged inner ring surface (Fig 5). Remarkably, this inner ring surface feature is conserved in SLT70 (and LtgA; Fig 5) which shares very little sequence conservation with Cj0843 apart from the C-domain (S2 Fig). The outer ring surface of these ring-shaped proteins shows a more random distribution of positive, negative, and neutral residues. This ensemble of positive charges on the inside of the ring of Cj0843 explains why the majority of sulfate and acetate ions, that co-crystallized at 200mM concentration in both crystal forms, were observed along the inner ring of the doughnut-shaped Cj0843 protein (S6 Fig). Although predominantly positively charged along the inner ring, both Cj0843 and SLT70 (and LtgA) contain a small negatively charged cavity within the central pit. This localized electrostatic exception is due to the presence of the active site residues E390 and E498 in Cj0843 and the equivalent glutamic acids in SLT70 and LtgA (Fig 5). Overall, the conservation of the positively charged electrostatic surface in the inner rings of Cj0843 and SLT70 suggests a potential functional role in both proteins. A likely role is to attract the negatively charged carboxyl groups of the peptide PG sections in which a single tetrapeptide contains three carboxylates. To predict potential ligand binding pockets on Cj0843, we used SiteMap (Schrodinger) [40]. The active site groove (labeled ‘1’ in Fig 5) received the second highest score of 0.98 whereas the top site ranked site with a score of 1.08 is a strongly positively charged pocket (labeled ‘2’ in Fig 5). Most of the residues in this pocket 2 are amongst the most conserved in the epsilonproteobacteria LTs (S4 Fig) further pointing to an important role. In SLT70, this pocket 2 interacts with the peptide section of the PG GlcNAc-anhMurNAc-L-Ala-L-Ala fragment [24].

To probe the mechanistic importance of Cj0843’s doughnut shape and its charge distribution for recognizing the PG substrate, including in pocket 2, we carried out four sets of MD simulations. These sets of MD simulations have the PG strands modeled into the active site either (i) in a substrate-binding mode with either a deprotonated or (ii) protonated E390, (iii) a product-binding mode, or (iv) a more unbiased PG starting position situated mostly outside the central hole of Cj0843. In the substrate PG, the GlcNAc and 1,6-anhydro-MurNAc units on the reducing end that are to be cleaved off are numbered as +1 and +2; the adjacent saccharide moieties prior to the cleaved bond are numbered -1 through -8. In the product PG strand, the +1 and +2 moieties are not present, and the -1 unit has become a 1,6-anhydro-MurNAc termini. The starting position for the substrate-binding mode was obtained by modeling a 5 disaccharide unit PG strand into the active site with the GlcNAc-2 in the same position as the GlcNAc in the bulgecin A complex structure. The ring of the PG MurNAc-1 with its hydroxyl-C6-methyl moiety was positioned similarly to the hydroxyl-methyl-pyrrolidine from the bulgecin A complex structure. The starting positions of the GlcNac+1 and 1,6-anhydro-MurNAc+2 were guided by the SLT70:1,6-anhydromurotripeptide complex [24]. The second set of substrate-bound simulations was carried out with the only difference being protonation of E390. For the third set of simulations, the starting position for the product-binding mode was acquired by modeling a 4 disaccharide unit PG strand in the active site with a similar starting position for the GlcNAc-2 and 1,6-anhydroMurNAc-1 moieties as in the substrate simulations yet without the (cleaved) +1 and +2 saccharide moieties. MD simulations for both the substrate and product PG strands were carried out in quadruplicates each with slightly different initial orientations/conformations of both termini of the PG strand. In the final set of MD simulations, the starting PG positions for all moieties but the terminal tetrapeptide were situated outside the central hole. Overall, the root-mean-square-deviation (RMSD) for main chain atoms over time averaged around 3Å (Figures A-D in S7 Fig). The largest root-mean-square-fluctuations (RMSF) occurred in the NU-loop and adjacent helices in the U-domain (Figures E-I in S7 Fig).

### MD of PG in substrate-binding mode

**Fig 6:**
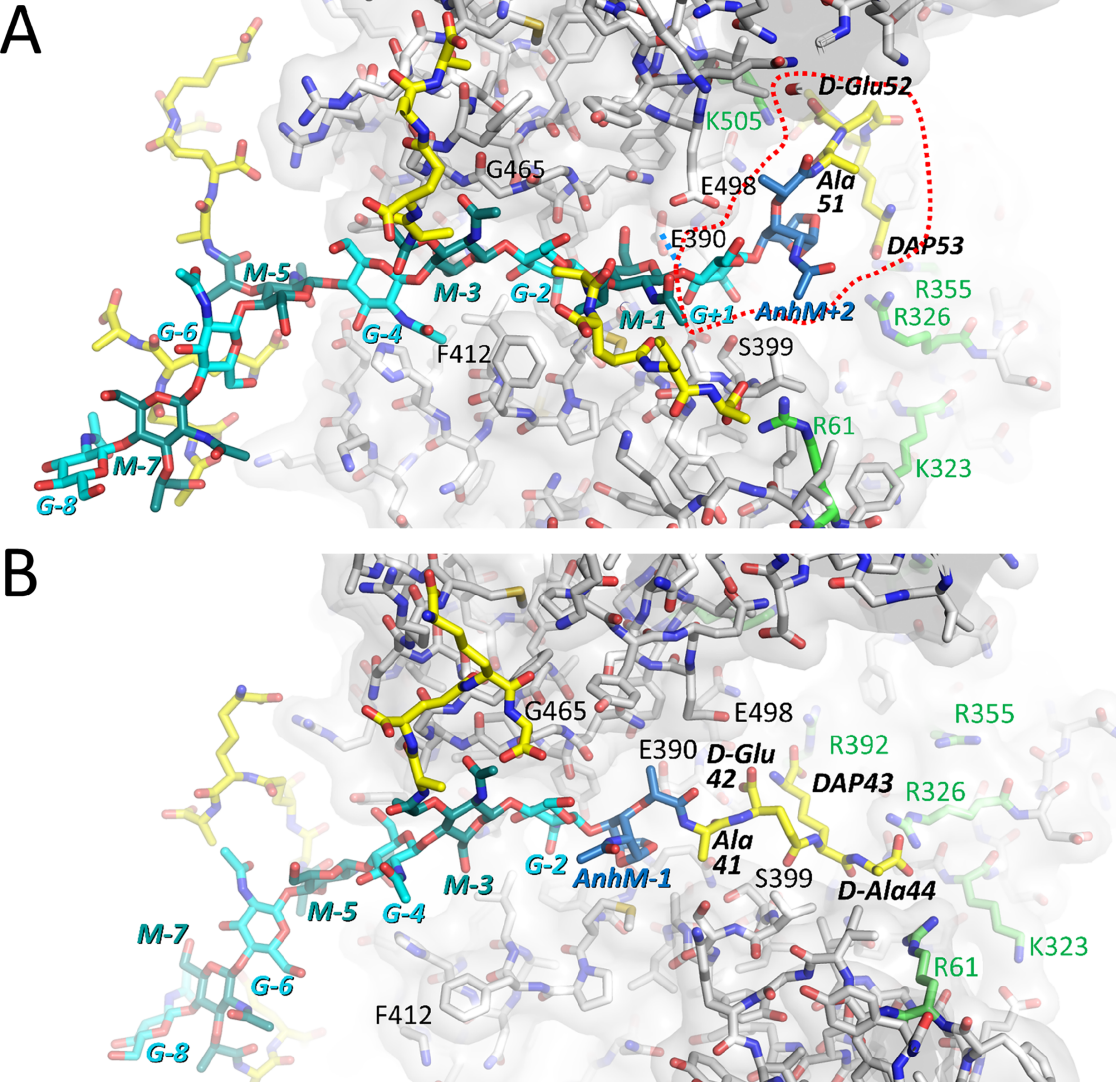
Molecular snapshots of PG strands in the Cj0843 active site modeled in substrate and product-binding modes. A A 5 disaccharide unit PG strand modeled as a substrate in the active site. A dashed red line highlights the terminal disaccharide peptide unit to be cleaved off. The PG tetrapeptide sections are shown with yellow carbon atoms. The PG GlcNAc (‘G’), MurNAc (‘M’), and 1,6-anhydroMurNAc (‘AnhM’) moieties are shown with light blue, blue/green, dark blue carbon atoms, respectively. Arg and Lys residues in the pocket 2 found to interact with the carboxyl moieties of the tetrapeptide are shown with light green carbon atoms. Key active site residues are labeled including the catalytic E390. B A 4 disaccharide unit PG strand modeled as a product in the active site.

The two sets of MD simulations of the PG in the substrate-binding mode (Fig 6A) were analyzed in detail in both the pocket 2 region (S8 Fig) and in the adjacent active site groove (S9 Fig). These analyses indicate that a number of hydrogen bonds persist throughout most of the independent 60-100ns MD runs: an internal hydrogen bond between the nitrogen of Ala51 of the terminal PG peptide moiety and the O6 atom of 1,6-anhydroMurNAc+2 (Figure B and G in S8 Fig), a hydrogen bond of the GlcNAc+1 with S399 and backbone oxygen of E390 (Figures B, C, G, and H in S8 Fig), a hydrogen bond of the O6 atom of MurNAc-1 with the catalytic E390 (Figures C and H in S8 Fig), hydrogen bonds of the main chain atoms of Y463 and M410 with the *N*-acetyl group of GlcNAc-2 (Figures B and G in S9 Fig), and hydrogen bonds of MurNAc-3 with main chain nitrogens of G468 and F469 (Figures D and I in S9 Fig).

**Fig 7:**
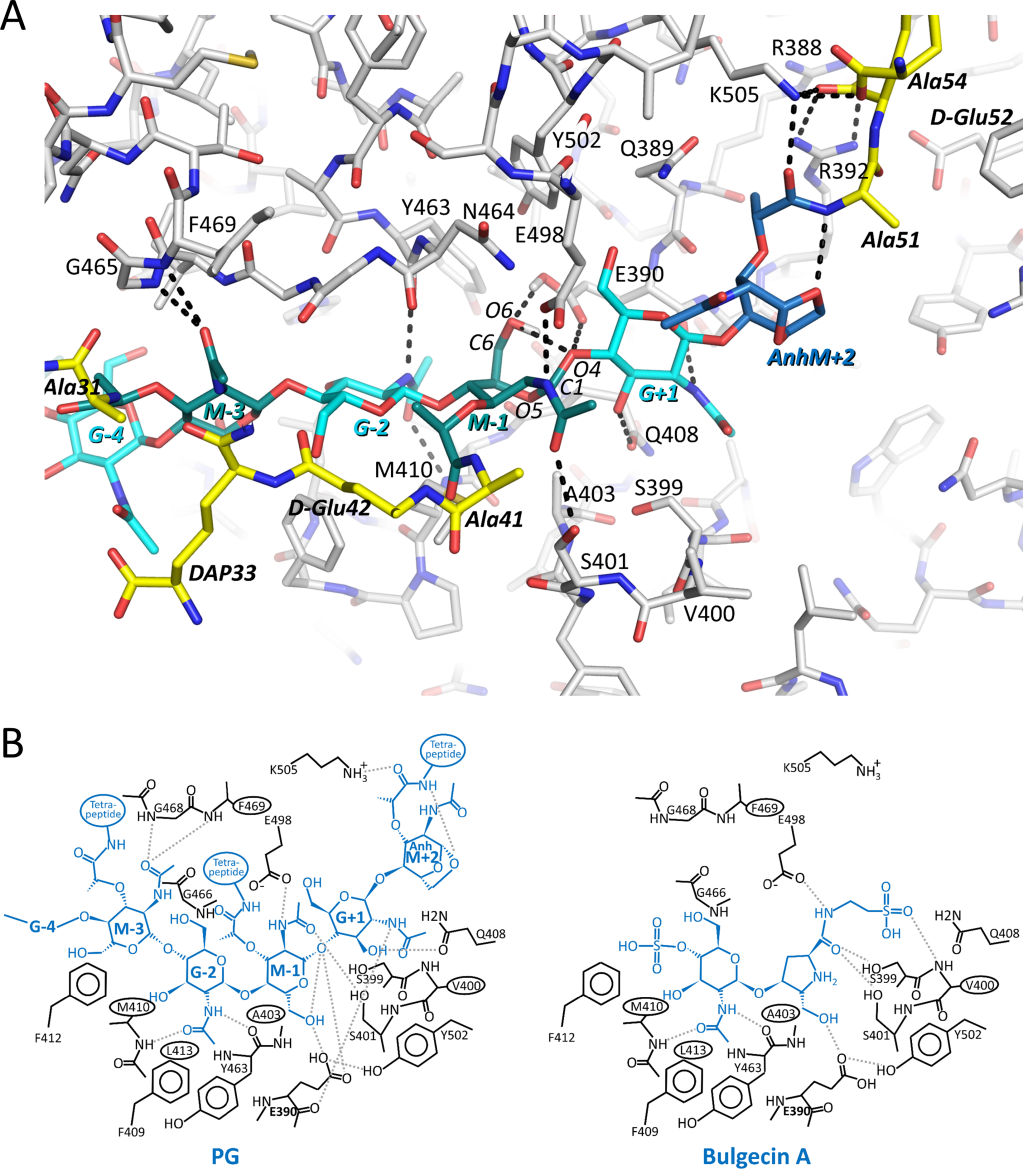
MD snapshot of the 5 disaccharide unit PG strand in the active site of Cj0843 in the substrate-binding mode. A The snapshot is at time point 57.82 ns of the protonated E390 MD run 3. The coloring and labeling of the PG strand are the same as in Fig 6. Hydrogen bonds are depicted as dashed black lines. MurNAc-1 (‘*M-1’*) is observed to be in a boat conformation. Hydrogen atoms for PG and protein are not depicted except for the hydrogens on the protonated side chain of E390 and on the O6 atom of MurNAc-1. B Schematic diagram of the PG strand interactions in the active site of Cj0843 (left, from 57.82 ns time point) compared to the crystal structure of bulgecin A bound in the active site (right).

The most significant difference between the deprotonated and protonated E390 MD runs is the distance between the catalytic E390 carboxyl group and the O4 oxygen-containing glycosidic bond that E390 assists to undergo cleavage (between MurNAc-1 and GlcNAc+1; Figure A in S9 Fig). All 4 MD runs with the protonated E390 yielded a distance of just over 4Å between this O4 atom and the CD atom of deprotonated E390 carboxyl moiety (Figure H in S9 Fig). This short distance is only present in the last of the deprotonated E390 MD runs (up to ~75ns; Figure C in S9 Fig). Another difference is that all 4 MD runs with the protonated E390 showed instances where the MurNAc-1 ring adopts a boat conformation, with runs 2 and 3 more long-lived and runs 1 and 4 more fleeting (Figure H in S9 Fig). This boat conformation was tracked by measuring the distance between C1 and C6 atoms of MurNAC-1, which is ~3.1 and ~3.7Å for the boat and chair conformations, respectively. In contrast, this boat conformation was observed in only the fourth of the deprotonated E390 MD runs (Figure C in S9 Fig; the chair to boat conformational change occurred during the 900ps NPT run before the production run 4). This MurNAc-1 boat conformation results in its O6 hydroxyl being within hydrogen bonding distance to the O4 atom of GlcNAc+1 as shown in a 48.59ns time point snapshot of run 3 of the protonated MD run (Fig 7). The distance between the O6 MurNAc-1 atom and the CD atom of E390 is also more consistently conducive to hydrogen bonding of the O6 atom with the E390 carboxylate oxygens in the protonated E390 MD runs compared to the non-protonated runs (Figures C and H in S8 Fig). This hydrogen bonding network, as observed in the 48.59ns time point snapshot, involving E390, the O4 atom of GlcNAc+1, and the O6 atom of MurNAc-1 is just one observed possibility (Fig 7); future QM/MM analyses could pinpoint the precise hydrogen bonding pattern that occurs during catalysis. In the boat conformation, the acetamide moiety of MurNAc-1 can form hydrogen bonding interactions with the mostly conserved E498 and S401 residues (Fig 7). These PG:LT interactions involving E498 and S401 are similar as observed in the bulgecin A:LT complex (Figs 3B and 7B). Furthermore, GlcNAc+1’s nitrogen makes a hydrogen bond with the backbone oxygen of E390 (Fig 7). This hydrogen bond is analogous to what GlcNAc makes in the GlcNAc-MurNAc-D-Ala-D-Ala complex with SLT70 [24]. The other PG interactions in the run 3 snapshot, involving GlcNAc-2 and MurNAc-3 (Fig 7), are similar to those described above.

The three carboxylate moieties of the terminal tetrapeptide attached to 1,6-anhydroMurNAc+2 make electrostatic interactions within the positively charged pocket 2 on Cj0843. This pocket harbors 8 Arg or Lys residues; their distances to the carboxylates on the terminal PG tetrapeptide residues D-Glu52, DAP53, and D-Ala54 are plotted versus simulation time (Figures D-F and I-K in S8 Fig; S1 Video). During all 8 trajectories, a minimum of one salt bridge interaction is observed for the tetrapeptide with an additional second salt bridge being present most of the time. Occasionally, up to five salt bridge interactions are observed such as in run 2 of the deprotonated E390 run at around 60ns (Figures D, E, and F in S8 Fig). The Glu52 carboxylate favors the subpocket lined by R388, R392, and K505—making interactions with one or more of these residues throughout most of the trajectories. In addition to these PG interactions, we also monitored the width of the active site cleft as well as the variable distance between the α-helices that anchor the NU-loop allowing the circumference of the circular Cj0843 to vary. Furthermore, we monitored the NU-domain interface *via* the distance between A33 and I398, and measured the distance of the L-domain relative to the active site (Figures E and J in S9 Fig). Finally, we also monitored the distances of the individual PG disaccharide units and tetrapeptide sections with respect to the active site (Figures F and K in S9 Fig). The disaccharide units that are furthest from the active site show the largest distance variations due to limited interactions with Cj0843 being mostly outside its central hole; their attached tetrapeptide moieties show even larger distance fluctuations likely due to these being more flexible compared to the disaccharide units. These additional analyses also show that the active site width, both in the deprotonated and protonated E390 MD series, can occasionally expand. For example, such widening takes place at time points ~12ns and ~65ns in run 1 (Figure E in S9 Fig; deprotonated E390 MD series) with a concomitant loss of 1 or 2 hydrogen bonds involving the GlcNAc-2 *N*-acetyl group (Figure B in S9 Fig). In this run, the GlcNAc-2-MurNAc-1 disaccharide slips out of the active site from the 65ns time point onward (Figures B-C in S9 Fig). This latter event results in an active site widening, now partially empty, that is even more pronounced around the 86ns time point till the end of the run 1 simulation (Figure E in S9 Fig). Note that the MurNAc-3 interactions in the active site remained stable during this event (Figure D in S9 Fig). While the NU-domain interaction with the rest of Cj0843 remains intact, the distances between the helices α4 and α5 that span the NU-loop between them are remarkably variable allowing the doughnut-shaped ring of Cj0843 to contract and dilate (Figures E and J in S9 Fig). The L-domain contains three of the R/K residues, and its center of mass relative to the active site shows some modest fluctuations (Figures E and J in S9 Fig).

### MD of PG in product-binding mode

The 260ns MD simulations of the PG strand in the product-binding mode reveal similarities with the substrate-binding mode simulations (Fig 6B and S10 Fig). These similarities involve an internal hydrogen bond between the nitrogen of Ala41 of the terminal PG peptide moiety and the O6 atom of 1,6-anhydroMurNAc-1 (Figures A-B in S10 Fig), the hydrogen bonds of the main chain atoms of Y463 and M410 with the *N*-acetyl moiety of GlcNAc-2 (Figure G in S10 Fig), and hydrogen bonds of MurNAc-3 with main chain nitrogens of G468 and F469 (Figure H in S10 Fig). Additional relatively persistent hydrogen bond interactions involve the backbone oxygen of D-Glu42 with the backbone nitrogen of V400 (Figure C in S10 Fig) as well as the internal salt-bridge interaction of the carboxylate moiety of D-Glu42 and the amine group of DAP43 (occurring about 50% of the time), both belonging to the terminal PG peptide section (Figure B in S10 Fig). Interestingly, the terminal tetrapeptide section, now attached to 1,6-anhydroMurNAc-1, makes one or more salt-bridge interactions with the positively charged pocket 2 on Cj0843 virtually the entire length of the simulations (Figures D-F in S10 Fig and S2 Video). The terminal tetrapeptide residues DAP43 and D-Ala44 can make salt bridge interactions in pocket 2 whereas residue D-Glu42 does not as it cannot reach far enough. These observations indicate that the positive charged pocket 2 can interact with the terminal tetrapeptide carboxyl moieties of the PG when bound in a substrate-binding mode as well as the product-binding mode; this despite the latter having a substantially shorter reach as it lacks the cleaved off disaccharide PG unit. The product simulations also showed instances where the width of the active site can become enlarged, such as around 135ns in run 1 and 95ns in run 2 (Figure I in S10 Fig); this leads to a concomitant loss of the hydrogen bond between the GlcNAc-2 and the backbone nitrogen of M410 (Figure G in S10 Fig). The two helices that anchor the NU-loop also show considerable variability in their distance from each other during each of the runs (Figure I in S10 Fig) as also observed in the substrate MD runs. As in the substrate MD runs, the disaccharide units furthest from the active site show the largest distance variations due to their location being mostly outside the central hole (Figure J in S10 Fig).

### MD of PG in starting position outside central pore

**Fig 8:**
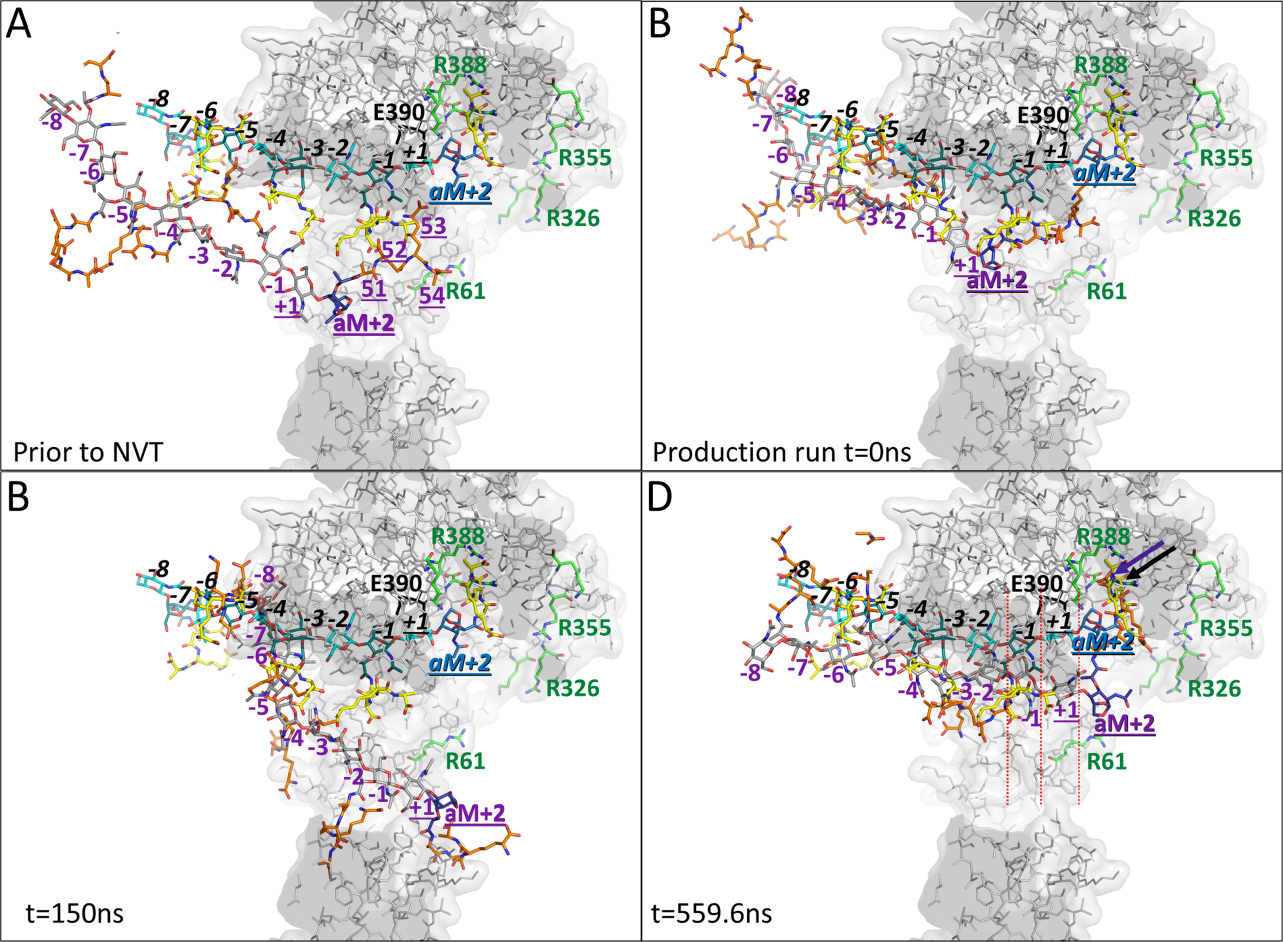
MD snapshots of the 5 disaccharide unit PG strand in the active site of Cj0843 as part of the 1μs simulation. A The starting position and starting conformation of the PG strand after minimization but before the NVT. The tetrapeptide moieties of the terminal disaccharide unit of the PG strand are labeled ‘51’ through ‘54’. The GlcNAc and MurNAc moieties are shown with grey carbon atoms whereas the tetrapeptide moieties are shown with orange colored carbon atoms; these PG moieties are labeled in purple. For reference, this and subsequent snapshots also contains a PG strand in the active site as obtained from the simulations with the PG in the substrate-binding mode (same binding mode and coloring as in Fig 6A yet with black italicized labels). The 1,6-anhydroMurNAc moiety is colored darker and labeled bold ‘aM+2’ for both PG strands in each panel. The labels of the moieties of the terminal disaccharide unit to be cleaved off in both the MD PG strand and the reference PG substrate strand are underlined. Residue E390 is labeled and shown in black sticks, and the Arg/Lys residues are shown with green carbon atoms as in Fig 6. The view is slabbed showing a side view that is roughly in a similar orientation as Fig 1B. B The PG strand after the NVT and NPT equilibration step but before the production MD run. C Snapshot at 150ns showing the PG has entered the pore but is bound unproductively distant from the active site E390. D Snapshot at 559.6ns showing that the terminal tetrapeptide section has reached the positively charged pocket 2 and makes similar carboxyl interactions as the equivalent tetrapeptide section in the comparison substrate modeled PG strand (arrows). At this latter time point, the glycan strand has approached the active site the closest within the entire 1μs simulation; the nitrogen of the *N*-acetyl moiety of GlcNAc-2 residue is within 7Å the Y463 main chain oxygen. Also, the anchoring in pocket 2 lined up the correct MurNAc-1 and GlcNAc+1 with respect to their equivalent moieties in the substrate-binding mode (red dashed lines) to facilitate cleavage of the terminal disaccharide PG unit pending the final approach to the active site groove.

To probe whether a PG strand could find its way to the active site groove and positively charged pocket 2 in a more unbiased manner, we positioned a 5 disaccharide unit PG strand such that only its terminal tetrapeptide moiety is within the central pore with the remainder of the PG strand positioned in the solvent region. We ran the simulations four times with starting orientations of the PG strand each differing by ~90° in rotation parallel to the central axis of the doughnut-shaped Cj0843. Of the four independent MD runs of 140ns each, two runs resulted in the PG strand entering the central hole whereas the other two runs did not. The two MD runs that showed the PG successfully entering the inner pore were continued for total simulation times of 560ns and 1μs, respectively. In the longest MD run, the PG strand approached and interacted with the positively charged peptide pocket 2 *via* its terminal tetrapeptide; this is similar to the interactions observed in the substrate and product MD simulations (Fig 8, S11 Fig, and S3 Video). This tetrapeptide pocket 2 interaction formed at ~310ns and persisted for the remainder of the simulation; it appears to anchor and thus restrict the movement of the entire PG strand (Figures A-C in S11 Fig). While this pocket 2 anchoring brought the PG saccharide strand in closer proximity to the active site, the PG saccharide strand did not enter into the active site groove during the 1μs simulation. Nevertheless, the anchoring of the PG strand caused the -2, -1, +1, and +2 saccharide units to be aligned with these moieties’ positions in the substrate-binding mode except for a final upward approach towards the active site groove (Fig 8D). Though not observed within the 1μs simulation, this final movement required for productive substrate-binding is relatively minor as the GlcNAc-2 *N*-acetyl nitrogen atom was within 7.0Å of the backbone oxygen of Y463 around the 560ns time point (Fig 8D and Figure E in S11 Fig); this distance is generally around 3Å to facilitate the critical hydrogen bond interaction that occurs both in the product and substrate-binding modes (Figs 6 and 7, Figures B and G in S9 Fig, and Figure G in S10 Fig). Prolonged, extended simulations will likely capture the completion of this PG approach into the active site groove but are beyond the scope of this work.

The width of the (empty) active site groove was more expanded and more variable in the 1μs simulation with the PG placed outside compared to the substrate and product-binding mode simulations in which the active site groove was occupied by the PG strand (Figure F in S11 Fig and S12 Fig). A similar active site groove narrowing was observed upon bulgecin A binding (Fig 3C). As with the previous simulations, the doughnut-shaped ring of Cj0843 was observed to contract and dilate as allowed by the stretching and compacting of the flexible NU-loop; this lead to fluctuations in distance between the α4 helix of the NU-domain and the α5 helix of the U-domain (Figure I in S7 Fig and Figure F in S11 Fig).

## Discussion

The crystal structure of the soluble LT Cj0843 from *C*. *jejuni* revealed an unusual doughnut-shaped structure containing four domains: NU-, U-, L-, and C-domains (Fig 1). Its closest known structural homolog is *E*. *coli* SLT70 [8, 24, 25], despite Cj0843 being about 100 residues shorter and having only significant sequence similarity in the catalytic C-domain (Fig 2 and S2 Fig). Cj0843 nevertheless has a similar sized central hole which is formed by a flexible linker-NU-domain configuration that completes the protein’s ring shape (Fig 1). Sequence analysis of other epsilonproteobacteria family LT members show that this ring-completing NU-domain interface is conserved (S2 and S4 Figs), suggesting an important functional role. The interface between the NU-domain and the L-/C-domains is only 678Å^2^, on the smaller end of observed protein-protein interfaces; such an interface corresponds to a projected weak affinity of around 10μM [42]. However, the NU-domain is physically tethered to the U-domain *via* a ten residue linker making the local effective concentration of this NU-domain very high; we calculate this local concentration to be ~5mM (1 molecule in a volume of a sphere with a diameter of (10+1) residues x 3.8 Å average Cα-Cα distance). This calculation indicates that despite its anticipated low inherent NU-domain affinity for the rest of Cj0843, its high local concentration will keep the interface intact for the majority of the time thereby maintaining the protein’s circular shape. Even during the 1μs MD simulation, the NU-domain interaction with C- and L-domains remained intact (Figure F in S11 Fig). One potential mechanistic purpose for the soluble LTs ring-shaped architecture is to facilitate processivity of PG cleavage as previously proposed based on their circular shape [43]. An additional purpose could be preventing degradation of critical internal PG sections by allowing only non- or minimally cross-linked end sections of PG strands to fit through the central hole. Such non- or minimally cross-linked sections can arise as a result PBP-directed antibiotics which inhibit the transpeptidase reaction yet leave the transglycosylase reaction intact causing structural defects in the PG layer, a defect that can be corrected by LT activity [44].

The non-catalytic domains of Cj0843 have distant structural similarity with protein domains involved in protein:protein interactions with each of the obtained DALI Z-scores scores being substantially above the 2.0 cut-off indicating significant similarities [45]. Both the L- and U-domain share structural similarity to bacterial chaperone proteins involved in type III secretion systems, AscG and IpgC, respectively. Whether coincidental or due to either divergent or convergent evolution, it is interesting to note that *C*. *jejuni* is naturally competent for transformation [46] which could facilitate a divergent evolutionary mechanism. An additional connection is that LTs function in type III secretion systems in many Gram-negative bacteria [47, 48] and are essential for virulence. There is also structural similarity between the L- and U-domains as both domains contain a TPR-like fold which is a common protein-protein interaction motif [49, 50]; such a motif seems well suited for the cumbersome purpose of generating a complicated doughnut-shaped structure *via* inter-domain interactions. The L-domain also forms a vital part of the positively charged pocket 2 to which it provides three R/K residues; the MD simulations indicate that this pocket has a role in anchoring the terminal tetrapeptide moiety. Finally, also the NU-domain shares structural similarity with a protein speculated to be involved in protein:protein interactions: the N-terminal domain of eIF2B, a domain that is postulated to be important for interacting with other proteins [51].

We showed that Cj0843 can bind to and is inhibited by bulgecin A (Figs 3 and 4 and Figures B-C in S3 Fig); the measured K_d_ for bulgecin binding was 210 μM ± 28 μM. Bulgecin A did however not result in complete inhibition of Cj0843 (Fig 4A). This partial inhibition could perhaps be due to relative modest binding affinity of bulgecin A as well as the likely processive nature of the reaction with the PG strand: once anchored into pocket 2, the PG strand stays in very close proximity to the active site groove thereby perhaps blocking bulgecin A entry while it continues to degrade the PG strand. The narrowing of the active site upon bulgecin A (Fig 3C) and PG binding (S12 Fig) is analogous to that previously observed for PG binding to SltB3 [27].

Although the catalytic rates for Cj0843 and SLT70 are not known, the k_cat_ for several other LTs has been determined. These k_cat_ values were measured to be 0.45, 2.52, and 19.9s^-1^ for *P*. *aeruginosa* MltB [52], SltB1 [53], SltB3 [27], respectively. Due to their similar active sites, it is likely that the Cj0843 reaction occurs on a similarly slow timescale. Obviously, our MD simulations with a maximal length of 1μs are not long enough to capture an entire lytic reaction cycle yet nevertheless likely captures key events that occur during the reaction. These events include the stable anchoring interactions of the negatively charged terminal tetrapeptide in pocket 2 in the substrate, product, and random starting position MD runs. The latter simulation showed that the PG aligns itself with the catalytic machinery for cleavage of the terminal disaccharide PG unit, thus promoting exolytic activity (Fig 8D). Additionally, we observed in both MD simulations and crystal structures, a stabilizing clamping or pinching motion of the active site upon bulgecin A (Fig 3C) or PG strand binding (S8–S12 Figs). Key pinch points are the backbone atoms of M410 and Y463 that form hydrogen bonds with the most deeply embedded portion of either bulgecin A or PG, the N-acetyl group of the GlcNAc. MD simulations showed that the PG bound active site undergoes breathing motions that allow an occasional widening of the active site and subsequent loss of these N-acetyl interactions and a partial PG release from the active site (S9 Fig, run 1). Furthermore, the MD simulations also showed that a protonated E390 leads to more catalytically conducive distances between the E390 carboxyl and O4 atom of the PG bond to be cleaved (Figure H in S9 Fig). Finally, the simulations also showed that the active site can promote the chair-to-boat conformational change of the MurNAc-1 moiety (Figures C and H in S9 Fig); the boat conformation is needed during one of the steps of catalysis to allow the O6 atom to nucleophilically attack the C1 atom to form the 1,6-anhydro-MurNAc end.

**Fig 9:**
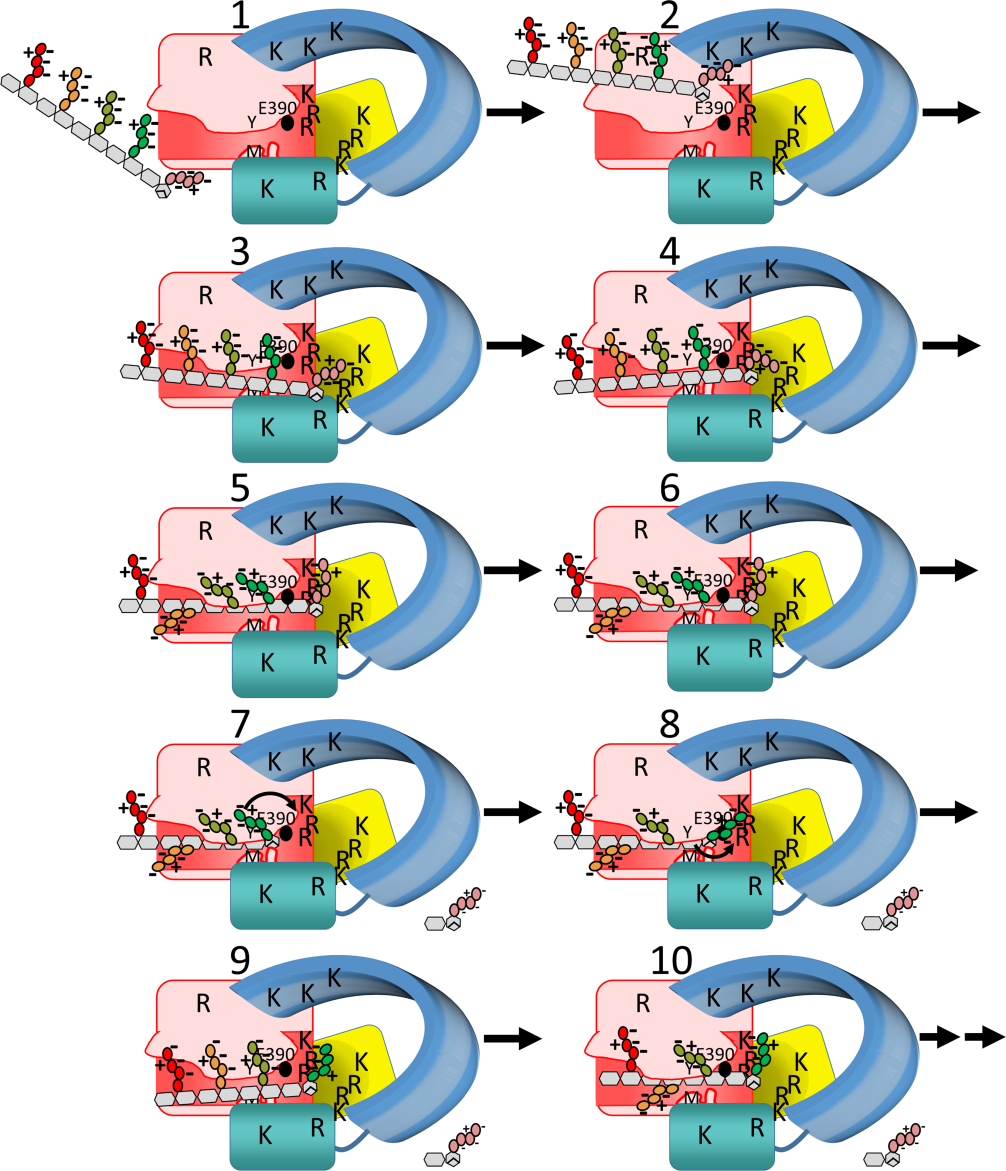
A proposed mechanism of PG hydrolysis by Cj0843. The colors of the domains of Cj0843 are as in Fig 1. The 8 R/K residues in pocket 2 are indicated, and several additional R/K labels are drawn for illustrative purposes. In addition to E390 (black sphere), M410 and Y463 are labeled ‘M’ and ‘Y’, respectively. A narrowing of the active site groove is depicted in states 6–8 with an accompanying shift of the flanking NU-domain. The boat conformation of MurNAc-1 is drawn in state 6. The tetrapeptide sections of the 5 PG disaccharide units are colored as in Figures F and K in S9 Fig and S3 Video.

Based on our above results, we propose the following events to occur during the hydrolysis of a PG strand in the active site of Cj0843 (Fig 9). This enzyme serves as a model for other epsilonproteobacterial LTs (S2 Fig), gammaproteobacterial LTs (such as SLT70 and Slts from *Pseudomonas aeruginosa* and *Stenotrophomonas maltophilia*; [18]), and betaproteobacterial LTs (such as LtgA; Fig 5):

1. *Electrostatic steering*: The PG 1,6-anhydro-MurNAc terminus of a non- or minimally-cross-linked section of a PG strand can fit and enter the central hole attracted by the positively charged inner surface (state 2 Fig 9).
2. *Terminal tetrapeptide homing to pocket 2*: The negatively charged tetrapeptide moieties of the PG strand will interact transiently with the positively charged patches present along the mostly convex surface of Cj0843 until the terminal tetrapeptide finds the positively charged pocket 2. The concave positively charged pocket, with 8 R/K residues available for multivalent interactions with the three tetrapeptide carboxyl moieties, is likely to form relatively strong substrate interactions due to avidity (state 3 Fig 9).
3. *Aligning PG strand and bond to be cleaved*: As this tetrapeptide of the 1,6-anhydroMurNAc becomes initially anchored in pocket 2 (in particular utilizing R388, R392, and K505 with R388 being 12.5Å from E390), it limits the movement of the entire PG strand and allows the PG strand to scan positions near the active site more efficiently (state 4 Fig 9). The anchoring and accompanying distance considerations likely also provide a molecular ruler that favors the alignment of the -1 and +1 saccharides with the catalytic machinery, such as to promote exolytic activity *via* cleaving off the terminal disaccharide PG unit.
4. *PG strand enters active site groove*: The PG saccharide strand next enters the active site groove (state 5 Fig 9) with a subsequent narrowing of this groove due to the multiple PG active site interactions including with M410 and Y463 (state 6 Fig 9). The -1 and +1 saccharide moieties, and flanking groups, are held in their respective positions in part by the terminal Glu52 residue interacting with the R388, R392, and K505 containing subpocket. The boat conformation of MurNAc-1 is hypothesized to be needed for the cyclization step after the protonated E390-assisted cleavage of the bond between MurNAc-1 and GlcNAc+1 [8]. MD simulations indicate that transition to the boat conformation can also occur before cleavage, making the two halves of the reaction more concerted.
5. *PG strand cleavage and product formation*: E390-assisted PG bond cleavage leads to release of the terminal disaccharide unit (state 7 Fig 9). After this bond cleavage, the second half of the reaction takes place: MurNAc, in the boat conformation, cyclizes to form the terminal 1,6-anhydroMurNAc-1 to complete the product formation. This step involves a nucleophilic attack of the C1 oxocarbonium intermediate by the O6 atom after proton abstraction by E390 [8, 18]. This step is likely aided by the observed interactions of the conserved E498 with the nitrogen of the acetamide moiety of the MurNAc-1 and the S401 interaction with the oxygen of this acetamide group. Analogous interactions were postulated for SLT70 to occur at this step [8] and are also observed in the bulgecin A complex for its equivalent N-acetyl moiety (Fig 3B). The anhydroMurNAc has a low affinity for the protein [24], resulting in the release of the cleaved disaccharide-tetrapeptide from the active site and pocket 2 area.
6. *New (product) tetrapeptide termini swings over to pocket 2*: The tetrapeptide moiety of the new terminus, 1,6-anhydroMurNAc-1, can swing over to interact with the positively charged pocket 2 *via* its terminal two carboxylates (state 8 Fig 9).
7. *Product PG strand dislodgement from active site groove*: While the product PG is now newly anchored to pocket 2, the saccharide strand can be dislodged from the active site groove without the PG strand wandering away from the active site region. This PG dislodgment from the groove can occur when the active site is occasionally widening leading to loss of the Y463/M410 hydrogen bonds while the tetrapeptide sections are concomitantly pulling on the PG strand (state 9 Fig 9). The terminal tetrapeptide can subsequently enhance its electrostatic interactions in pocket 2 using all three of its carboxylates with a likely vital role for the second PG amino acid carboxylate interacting with the R388, R392, and K505 sub-pocket (Fig 7). This interaction is similar to the equivalent Ala carboxyl moiety in the SLT70 GlcNAc-MurNAc-D-Ala-Ala complex interacting with the conserved SLT70 R476 [24](S2 Fig).
8. *Pocket 2-aided ratcheting of PG strand to facilitate processivity of next exolytic cleavage*: While enhancing the pocket 2 interactions with all of its three terminal carboxyl moieties, the PG strand slides deeper through the central hole such that the next terminal disaccharide unit to be cleaved approaches the active site E390. The PG strand will now enter the active site groove again to start the next lytic transglycosylase reaction cycle (state 10 Fig 9). This processivity may continue until the PG strand is degraded or a PG section is encountered that is too cross-linked to fit through the central hole of Cj0843.

We note that these postulated mechanistic steps are likely the dominant catalytic path, but we anticipate that exceptions at specific steps can occur as a) the mostly exolytic SLT70 was found to also have some minor endolytic activity, and b) the final cyclization step generating the 1,6-anhydroMurNAc can occasionally be skipped [9]. The importance of the tetrapeptide moieties in several of the steps in the above mechanism explains why SLT70 was found not to hydrolyze isolated poly-GlcNAc-MurNAc [54]. A postulated key role for the second amino acid (i.e., Glu52) in the tetrapeptide PG degradation is consistent with the observed PG peptide lengths. As the length of the peptide moieties in PG can vary from penta-, tetra-, tri-, and dimeric in *C*. *jejuni* [55] and other bacteria, even a PG with a minimal dipeptide length can thus likely be degraded. This degradation would be facilitated by binding the key carboxylate of the second residue with the conserved R388, R392, and K505 residues.

The central hole of the Cj0843, SLT70, and LtgA is much more substantial than that in SLTB3 (Figs 2 and 5) with the size of the pore being much larger than a single PG strand. This observation suggests that additional mechanism(s) act to promote processivity, such as to prevent a single PG strand from exiting prematurely after a single cleavage event. Such a mechanism might be the postulated ratchet mechanism in Fig 9 that enhances the mostly exolytic nature of this class of doughnut-shaped LTs and processivity in degrading PG strands. Future studies will include QM/MM dynamics to simulate detailed mechanistic steps including proton abstraction events during the reaction as well as to probe whether the hole of the larger LTs can accommodate two cross-linked PG strands.

## Materials and methods

### Protein expression and purification

The Cj0843 expression vector (Cj0843-pET28b) was used for expression as previously described [19]. The protein was expressed by inoculating LB with overnight transformations of BL21 star cells (ThermoFisher) with Cj0843-pET28b. Once the culture reached an OD600 of 0.5, the protein expression was induced with 0.5mM IPTG. Four hours after induction, the cultures were harvested by centrifugation and resuspended in lysis buffer containing 50mM sodium phosphate pH 8.0, 300 mM sodium chloride, and 20mM imidazole. The cells were lysed using a microfluidizer; 5 mM MgCl_2_ and 5 mM ATP was added to the supernatant to remove bound chaperones. After centrifugation, pre-equilibrated Ni-NTA beads (Qiagen) were added to the supernatant followed by rocking at 4°C. The beads with bound proteins were washed with the lysis buffer and the protein was eluted with 50 mM sodium phosphate pH 8.0, 300 mM sodium chloride, and 300mM imidazole. Glycerol was added to the eluate (final concentration 10%) which was flash frozen in liquid nitrogen and stored at -80°C until further use. The protein was further purified using gel filtration Superdex 200 10/300 column (GE Life Sciences) pre-equilibrated with 1x PBS buffer. The protein eluted as a monomer and was determined to be >95% pure by SDS-PAGE.

The selenomethionine derivatized Cj0843 was expressed using the van Duyne protocol [56] and purified using the same protocol described above except that the buffers also included 2mM β-mercaptoethanol.

### Crystallization, data collection, and refinement

The protein buffer was exchanged to 10 mM Hepes pH 7.5 using a 30KDa MWCO concentrator (Millipore). Native and selenomethionine derivatized Cj0843 was crystallized using 0.2 M lithium sulfate, 0.1 M Tris pH 8.0, and 39% PEG 400. The thin hexagonal crystals were frozen in liquid nitrogen without additional cryo-protection. Multi-wavelength anomalous dispersion (MAD) diffraction data were collected at the Selenium K-edge absorption peak (0.9791Å), inflection (0.9793Å), and remote wavelengths (0.9116Å; Table 1). The P3_1_21 space group data were processed using Autoxds (A. Gonzalez and Y. Tsai, SSRL) [57] and the crystallographic phases were solved using Autosol [58](Table 1). The initial model generated by Autosol was further rebuilt using Coot [59] and refined against a higher resolution 1.88Å native dataset of Cj0843 using Phenix [60] and Refmac [61]. The model contains residues 19–76 and 81–532 with residues 77–80 not modeled due to poor density. Cj0843 was also crystallized in a different space group, I23, for which crystals diffracted to 2.3Å (Table 1). After molecular replacement using Molrep [62] with the coordinates from the P3_1_21 structure, the subsequent refinement of this I23 space group structure yielded a model of Cj0843 containing residues 18–533. Crystals of Cj0843 in the P3_1_21 space group were also used for soaking experiments involving bulgecin A (2.4mM for 40 min) which yielded a 2.04Å structure with bulgecin A bound in the active site (Table 1). Topology and parameter files for bulgecin A for refinement in REFMAC were obtained using PRODRG [63]. Coordinates and structure factors of the native, native I23 space group, and bulgecin A complexed Cj0843 crystal structures have been deposited with the Protein Data Bank with accession numbers 6CF8, 6CF9, and 6CFC, respectively.

**Table 1:** Data collection, structure solution, and refinement statistics for Cj0843 data sets.

Data collection	Native	Native I23	Bulgecin A complex	Selenomethionine multi-wavelength anomalous diffraction		
Wavelength (Å)	0.97946	0.97940	0.97946	0.9791 (peak)	0.9793 (inflection)	0.9116 (remote)
Data range	38.82–1.87	38.09–2.28	50–2.04	38.8–2.45	38.73–2.47	38.64–2.47
Space group	P3121	I23	P3_1_21	P3_1_21	P3_1_21	P3_1_21
a = b, Å	74.64	178.67	74.29	73.56	73.44	73.29
c, Å	194.09	178.67	190.46	193.98	193.63	193.21
Completeness (outer shell)(%)	98.3 (80.9)	99.6 (97.5)	99.1 (99.6)	99.4 (94.8)	99.6 (96.3)	99.5 (95.8)
Unique reflections (outer shell)	51659 (2700)	42902 (6096)	39342 (3871)	23057 (2423)	22435 (2385)	22329 (2355)
Total number of observations (outer shell)	983855 (33284)	1731887 (227827)	224186	458457 (47762)	430769 (35614)	437577 (45042)
Average multiplicity (outer shell)	19.0 (12.3)	40.4 (37.4)	5.7 (5.2)	19.9 (19.7)	19.2 (14.9)	19.6 (19.1)
Mean I/sd(I) (outer shell)	16.3 (2.1)	21.5 (2.2)	11.6 (2.0)	16.7 (2.8)	16.4 (1.8)	15.1 (2.0)
R_merge_	0.092 (0.510)	0.111 (2.533)	0.134 (0.647)	0.168 (1.351)	0.153 (1.619)	0.172 (1.769)
**Phasing**						
Resolution				38.8–2.48		
Number of Se sites				13		
Mean FOM				0.56		
**Refinement**						
R _work_	0.191	0.200	0.177			
R _free_	0.242	0.232	0.220			
Resolution (Å)	38.82–1.87	38.82–2.29	50–2.04			
Ligands	7 sulfate ions	1 PEG 2 acetate ions	1 bulgecin A 9 sulfate ions			
Number of water molecules	409	224	291			
RMSD bong length, (Å)	0.009	0.009	0.010			
RMSD bond angles, (°)	1.29	1.29	1.31			

### Differential scanning fluorimetry

A concentration of 5μM Cj0843 with 10x SYPRO orange fluorescent dye in 1xPBS buffer was used to determine the melting temperature using a Biorad CFX96 instrument. The temperature was ramped up from 20 to 95°C. Standard deviations for the triplicate experiments were found to be below 0.3°C. For obtaining a quantitative measure of bulgecin A affinity for Cj0843, duplicate DSF measurements were carried out with varying concentrations of bulgecin A (5 μM– 640 μM). The DSF data was analyzed using GraphPad Prism (Graphpad Software, La Jolla, CA) using the Single Site Binding formula [64].

### *C*. *jejuni* growth assay

An *in vitro* growth assay was performed in a microtiter plate to determine if bulgecin A can potentiate the efficacy of ampicillin (Amp) against β-lactam resistant *C*. *jejuni*. Briefly, the Amp resistant *C*. *jejuni* strain JL974 [19, 65] was grown to exponential phase and was inoculated in Müller-Hinton (MH) broth (200 μL/well) or MH broth supplemented with Amp (128 μg/mL, sublethal concentration), bulgecin A (100 μg/mL), or Amp (128 μg/mL) plus bulgecin A (100 μg/mL). Duplicate wells were used for each specific growth medium. The cultures were incubated at 42°C under microaerophilic condition (85% N2, 10% CO2, 5% O2) for 30 h. During the incubation, 20 μL of samples were taken at different time points (0, 6, 24 and 30 h), serially diluted, and plated on MH agar for enumeration of *Campylobacter* colonies in each sample. The log_10_-transformed colony-forming units (CFU) for each sample was used to compare the growth of the *C*. *jejuni* strain in different media.

### Lytic transglycosylase turbidity activity assay

The turbidimetric assay to monitor LT activity was carried out similarly as previously described [19]. PG substrate was isolated from *C*. *jejuni* 81–176 derivative using chloroform extraction method [19] with slight modification. In brief, the overnight grown Cj0843 mutant of *C*. *jejuni* 81–176 from 10 Mueller-Hinton plates were harvested and suspended in 40 ml 0.5x PBS (pH7.4). 3 Ml of chloroform was subsequently added to the suspension, and the mixture was gently rocked at room temperature for 30min. Subsequently, the chloroform-extracted crude PG cell walls were pelleted by centrifugation and washed once with 1x PBS (pH7.4). Finally, the crude PG was resuspended in 40 mL of 1x PBS. When turbidity activity measurements were performed, 1μM Cj0843 enzyme is added to the turbid PG cell wall suspension in PBS buffer at 20°C; the reaction volume is 75μl, and the absorbance at 450 nm was measured using a Molecular Devices FilterMax F5 apparatus. The 4.5hr assay was carried out at room temperature and performed in triplicate.

### Molecular dynamics simulations

The Cj0843 protein coordinates and all crystallographic water molecules from the I23 space group were selected as starting coordinates for MD simulations as this structure contains the entire NU-loop/linker. The addition of protons and histidine protonation state assignments were carried out using Maestro/Schrodinger Protein Preparation Wizard (Schrodinger, LCC). The VMD software was utilized to place the protein in a rectangular box of water molecules extending 12.5Å in each direction from the protein surface (typical box of around 112 Å x 103 Å x 103 Å). Sodium and chloride ions were added to neutralize the system as well to set the concentration to 0.15M NaCl [66]. NAMD was used for MD simulations with the protein using the CHARMM force field parameters [67]. The CHARMM parameters for the PG were obtained from Gumbart et al [68] with modifications to include the 1,6-anhydro-MurNac termini. Regarding the PG substrate, tetramer peptide L-Ala-D-iGlu-*meso*-Dap-D-Ala was used as it is the predominant PG peptide unit in *C*. *jejuni* [55]. The starting conformations of the GlcNAc-MurNAc-tetrapeptide was guided by PDBid coordinates 3d2y, 5ctv, 4gvi, 2cb3, 1qte, and 3td5 containing these moieties. To obtain a starting conformation for modeling the entire PG strands near Cj0843, 4 disaccharide unit and 5 disaccharide unit (i.e., GlcNAc-MurNAc-tetrapeptide) PG strands were subjected to MD simulations by themselves for at least 8ns under similar conditions as for the protein. Bonds with hydrogen atoms were kept rigid using the SHAKE algorithm, and the time-step was 2fs. The system was first minimized for 3,000 steps and equilibrated for 2ps at 300K and an additional 100ps at 310K using NVT (constant number of particles, volume, and temperature). This step was followed by an equilibration NPT (= constant number of particles, pressure, and temperature) molecular dynamic run for 900ps at 310K and 1.01325 bar. After this, the production MD runs were carried out for time periods of 60ns up to 1μs at 310K. The switching function for treating van der Waals interactions was applied between 10 and 12Å. Electrostatic interactions were treated using the particle mesh Ewald summation (PME) with a grid spacing of 1.0Å. Four sets of MD simulations were carried out with Cj0843 with a PG strand comprised of either 4 or 5 disaccharide units; the terminal MurNAc on the reducing end was modeled as a 1,6-anhydro-MurNAc (Table 2). One set of MD simulations had the 5 disaccharide unit PG strand modeled in a substrate-binding mode with a deprotonated catalytic E390. The second set was identical except that the E390 was protonated (a protonated catalytic glutamic acid residue is postulated to aid in the mechanism of LTs [8, 18]). The third set of MD simulations had the 4 disaccharide PG strand modeled as the product in the active site. The fourth set of MD simulations had a 5 saccharide PG strand modeled in a more unbiased starting position situated mostly outside the central hole; this starting position had only the 1,6-anhydro-MurNAc end partially enter the hole in the mechanistically productive entry direction. Each set of MD simulations was comprised of 4 independent runs. Snapshots of the MD productions runs were saved every 10ps for subsequent analysis using the VMD software.

**Table 2:** Overview of different molecular dynamics simulations carried out for Cj0843 with PG.

PG starting position	PG length (# disaccharide-tetrapeptide units)	Length MD runs (ns)	# of independent runs	Variations in separate runs regarding starting position/ conformations of PG
PG in substrate- binding mode	5	100	4	G-2, M-1, G+1, AnhM+2 moieties modeled as guided by bulgecin A complex and related LT complex structures; other glycan and tetrapeptide moieties positions varied
PG in substrate- binding mode with protonated E390	5	60	4	Same as above
PG in product-binding mode	4	260	4	G-2 and AnhM-1 modeled as guided by bulgecin A complex; other glycan and tetrapeptide moieties positions varied
PG positioned in random starting position	5	140, 140, 560, and 1,000	4	Extended PG strand positioned mostly outside central pore (only terminal tetrapeptide of AnhM+2 is in center of the hole). Each run had a different PG starting orientation varied by a 90° rotation along the central axis of the pore

## Supporting information

S1 Fig Glycosylation sites mapped onto the Cj0843 structureCj0843. Native glycosylation sites N99, N175, N329, 376 are shown in spheres with grey carbon atoms. Catalytic E390 is shown in black spheres. The view is related to Fig 1B *via* a 90° rotation around the horizontal central axis. Domain colors are same as in Fig 1.
(TIF)

S2 Fig Sequence alignment of Cj0843 with LTs from other epsilonproteobacteria and *E*. *coli* SLT70. The alignment contains homologous LTs from *Helicobacter pylori*, *Epsilonproteobacteria bacterium*, *Sulfurovum lithotrophicum*, *Nitratiruptor* sp. SB155-2, *Lebetimonas* sp. JH292, and SLT70 from *E*. *coli*. The underlined SLT70 residues are in structurally equivalent positions in Cj0843. Residues are color-coded based on conservation; red residues are identical to the Cj0843, residues in orange are similar. The yellow boxed residues show the additional L-domain helix in SLT70. The C87-C102 disulfide bond is indicated in purple line. Blue boxes highlight residues at the interface between the NU-domain and the rest of Cj0843. Residues interacting either directly or indirectly (water-mediated) with bulgecin A are highlighted with a black oval. The black box shows the conserved catalytic glutamic acid. Positively charged residues with a blue bar above them are situated in the positively charged pocket 2.
(TIF)

S3 Fig Melting temperature changes of Cj0843 upon reducing its disulfide bond and binding bulgecin A. (*A*) DSF measurements of Cj0843 in the absence or presence of 10mM DTT and 10mM β-mercaptoethanol.
(*B*) DSF measurements of Cj0843 in the absence or presence of 1.7mM bulgecin A; a 3% DMSO control is included.
(*C*) DSF measurements of Cj0843 with varying concentrations of bulgecin A. Experiments were carried out in duplicate (1.67% DMSO included in the assays).
(TIF)

S4 Fig Conserved residues plotted on the surface of Cj0843. The degree of sequence conservation in Epsilonproteobacteria is color-coded onto the surface of the protein. The NU-domain, with the NU-loop removed, was moved 15Å away to expose the residues at the interface of C-domain, L-domain, and NU-domain. The figure was generated using ConSurf, and residue conservation ranges from non-conserved (dark blue/cyan) to conserved (dark magenta). The left and right figure are different orientations such that their views expose both sides of the C-/L-domain–NU-domain interface each highlighted by a dotted oval. Also conserved is the active site region labeled ‘2’ encompassing pocket 2 and the active site groove.
(TIF)

S5 Fig Close-up stereo view of the NU-domain interface with the C- and L-domains of Cj0843. Color coding of the individual domains is as in Fig 1.
(TIF)

S6 Fig Crystallographically observed ligands bound to the inner ring/active site region in the three Cj0843 structures. The following molecules were observed with their locations labeled in parentheses: Bulgecin A (1 with carbon atoms in cyan), a PEG molecule (2) and acetate ions (3 and 8; with carbon atoms in green) from the I23 space group structure, and sulfate ions (3–8) from either the bulgecin A complex or P3_1_21 apo Cj0843 structure. Sites 3 to 7 are located in the positively charged pocket 2. The slabbed view into the active site is along an axis perpendicular to the axis of the doughnut-shaped Cj0843 similar to the view in Fig 3. The C-domain (red), L-domain (yellow), U-domain (blue), and NU-domain (blue/green) are shown with the surface patch comprised of atoms of E390 shown in black.
(TIF)

S7 Fig The RMSD versus simulation time and versus residue of the MD runs of Cj0843 with different starting positions of the PG strands. (*A*) 5 Disaccharide unit PG strand in the substrate-binding mode with deprotonated E390.
(*B*) Same as A but with protonated E390.
(*C*) 4 Disaccharide unit PG strand in the product-binding mode.
(*D*) 1μs MD simulation of 5 disaccharide PG strand with an unbiased starting position.
(*E*) RMSF per residue of the substrate MD runs with deprotonated E390.
(*F*) Same as E but with protonated E390.
(*G*) RMSF per residue of the product MD runs.
(*H*) RMSF per residue of the 1μs MD simulation of 5 disaccharide PG strand in an unbiased starting position. The runs 1, 2, 3, and 4 of the substrate and product MD simulations in A-C and E-G are colored blue, orange, grey, and yellow, respectively.
(*I*) Tube representation of Cj0843 at the end of the 1μs MD simulation color-coded by average RMSF per residue ranging from 0.5 to 5.0Å corresponding to blue-green-red, respectively. The Cj0843 orientation is similar as in Fig 1A.
(TIF)

S8 Fig Inter- and intra-molecular distances near pocket 2 of the PG strand in the substrate-binding mode MD runs. The catalytic E390 was either deprotonated (*B*-*F*) or protonated (*G*-*K*).
(*A*) Close-up view of pocket 2 area of the active site of Cj0843 with PG strand in substrate-binding mode. Color coding and orientation are the same as in Fig 6A except a more zoomed in view is shown. Distances that are monitored are shown as colored dashed lines and correspond to those in subsequent panels *B*, *C*, *G*, and *H*.
(*B*) Distances plotted versus time between the CD atom of PG Glu52 and NZ atom of PG DAP53 (blue), the *N*-acetyl nitrogen atom of GlcNAc+1 and the backbone O atom of E390 (red), the nitrogen atom of PG Ala51 and the O6 atom of AnhMurNAc+2 (magenta).
(*C*) Distances are shown for the O6 atom of GlcNAc+1 and the CD atom of E498 (black), O6 atom of MurNAc-1 and the CD atom of E390 (magenta), and the O3 atom of GlcNAc+1 and the OH atom of S399 (green).
(*D*) Distances of the carboxyl CD of PG Glu52 and the NZ atoms of indicated K residues or CZ atoms of indicated R residues.
(*E*) Same as (*D*) but with PG carboxyl atom CZ of DAP53.
(*F*) Same as (*D*) but with PG carboxyl C atom of Ala54.
(*G*)-(*K*) Same as (*B*)-(*F*) but with protonated E390 residue during the simulations.
(PDF)

S9 Fig Inter- and intra-molecular distances near active site groove of the PG strand in the substrate-binding mode MD runs. The catalytic E390 was either deprotonated (*B*-*F*) or protonated (*G*-*K*).
(*A*) Close-up view of active site groove of Cj0843 with PG strand in substrate-binding mode. Distances that are monitored are shown as colored dashed lines and correspond to those in subsequent panels *B*-*D* and *G*-*I*.
(*B*) Distances plotted versus time between the oxygen of the *N*-acetyl moiety of GlcNAc-2 and the backbone nitrogen of M410 (red) and between the nitrogen of the *N*-acetyl moiety of GlcNAc-2 and the backbone oxygen of Y463 (blue).
(*C*) Distances of the O4 atom of GlcNAc+1, belonging to the bond to be cleaved, and the CD atom of E390 (black), and between the C6 and C1 atoms of MurNAc-1 indicative of a chair or boat conformation (brown). A black arrow indicates instances where a boat conformation starts.
(*D*) Distances between the O3 atom of MurNAc-3 and the backbone nitrogen of F468 (yellow), the oxygen atom of the *N*-acetyl moiety of MurNAc-3 and the backbone nitrogen of F469 (pink), the oxygen atom of the *N*-acetyl moiety of MurNAc-3 and the backbone nitrogen of G468 (magenta), and the CZ atom of F412 and the center of mass of residues 463–467 (grey).
(*E*) Distances between the center of mass of the L-domain and the center of mass of residues 388–392 (brown), the center of mass of residues 398–403 & 409–413 with respect to the center of mass of residues 460–470 & 497–502 representing the width of the active site clef (light blue), center of mass of NU-domain helix α4 and center of mass U-domain helix α5 representing the distance across the flexible NU-loop (green), and the distance of the CA atom of NU-domain A33 and CA atom of C-domain I398 representing the distance across the NU-domain interface.
(*F*) Distances of the 5 PG disaccharide units (G*x*M*y*) and their respective tetrapeptide moieties (Pep*xx*-*yy*) with respect to the center of mass of active site residues 463–467.
(*G*)-(*K*) Same as (*B*)-(*F*) but with protonated E390 during MD runs.
(PDF)

S10 Fig Inter- and intra-molecular distances of the PG strand in the product-binding mode MD runs. (*A*) Close-up view of pocket 2 area of the active site of Cj0843 with PG strand in product-binding mode. Distances that are monitored are shown as colored dashed lines. The view and atom coloring are as in Fig 6B but a more zoomed in view is shown.
(*B*) Distances plotted versus time between the oxygen atom of PG Glu42 and the OH group of S399 (orange), the nitrogen atom of PG Ala41 and the O6 atom of AnhMurNAc-1 (red), and the CD atom of PG Glu42 and NZ atom of PG DAP43 (blue).
(*C*) Distances between the oxygen atom of PG Glu42 and the backbone nitrogen of V400 group of S399 (black), and the oxygen atom of the *N*-acetyl moiety of AnhMurNAc-1 and the OH group of S401 (magenta).
(*D*)-(*F*) Same as in Figures D-F in S8 Fig but now with the PG carboxylates from tetrapeptide residues 42–44, respectively.
(*G*) Same as in Figure B in S9 Fig.
(*H*)-(*I*) Same as in Figures D-E in S9 Fig.
(*J*) Same as Figure F in S9 Fig but now lacking the cleaved off terminal disaccharide PG unit.
(PDF)

S11 Fig Inter- and intra-molecular distances of the PG strand during the 1μs MD run with starting position outside hole of Cj0843. Distances plotted versus time. (*A*)-(*C*) Same as Figures D-F in S8 Fig. (*D*) Distances plotted versus time between the CD atom of PG Glu52 and NZ atom of PG DAP53 (blue), and the nitrogen atom of PG Ala51 and the O6 atom of AnhMurNAc+2 (red). (*E*) Distances between the oxygen of the *N*-acetyl moiety of GlcNAc-2 and the backbone nitrogen of M410 (red), the nitrogen of the *N*-acetyl moiety of GlcNAc-2 and the backbone oxygen of Y463 (blue), and distances of the O4 atom of GlcNAc+1, belonging to the bond to be cleaved, and the CD atom of E390 (black). (*F*) Same as in Figure E and S9 Fig.
(TIF)

S12 Fig Distance distributions of the active site width in the different MD simulations. The width of the active site is defined as the center of mass of residues 398–403 & 409–413 relative to the center of mass of residues 460–470 & 497–502. Twenty distance bins were plotted for the Cj0843 active site width as observed in the substrate-binding mode simulation (green), product-binding mode simulation (magenta), and the 1μs simulation in which the starting PG position was placed outside the central hole of Cj0843 (black).
(TIF)

S1 Video MD simulation of Cj0843 with PG in substrate-binding mode starting position. Representative section of the substrate-binding mode MD run simulation with a close-up view of the terminal tetrapeptide-1,6-anhydroMurNAc+2-containing section of the PG strand. The domains are colored as in Fig 1. The PG atoms are shown in a ball-and-stick model with the tetrapeptide carbon atoms colored yellow, and the saccharide carbon atoms colored cyan. The tetrapeptide amino acid carboxylates are labeled by their residue number (52–54). Selected protein atoms are shown in stick model with the carbon atoms of the 8 R/H residues colored cyan and the catalytic E390 atoms shown in black. Hydrogen bonds of the tetrapeptide carboxyl moieties with the R/K residues or with its amine-group are shown as black dashed lines (cut-off of 3.2Å). The video contains part of run 1 of the deprotonated E390 MD substrate simulation. A smoothing step of 2 was used for generating this video.
(MPEG)

S2 Video MD simulation of Cj0843 with PG in product-binding mode starting position. Representative section of the product-binding mode MD run 2 simulation with a close-up view of the terminal tetrapeptide-1,6-anhydroMurNAc-1-containing section of the PG strand. The tetrapeptide amino acid carboxylates are labeled by their residue number (42–44). The view and color coding are similar as in S1 Video.
(MPEG)

S3 Video MD simulation of Cj0843 with PG in distant starting position. MD simulation of the entire 1μs trajectory of a 5 disaccharide unit PG with a starting position in which only the terminal tetrapeptide moiety is inside the central hole. The PG strand is shown in solid spheres with the saccharide moieties colored by atom type with grey carbon atoms; the 5 tetrapeptide moieties starting at the terminal 1,6-anhydroMurNAc are colored light red, green, olive, orange, and red, respectively. The domain colors of Cj0843 are similar as in Fig 1. The catalytic E390 is shown as black spheres situated in the red C-domain. The disulfide bond in the blue U-domain is shown as yellow spheres.
(MPEG)

S1 Report Validation report for crystal structure PDB reference 6CF8 from protein data bank. (PDF)

S2 Report Validation report for crystal structure PDB reference 6CF9 from protein data bank. (PDF)

S3 Report Validation report for crystal structure PDB reference 6CFC from protein data bank. (PDF)
