# Supplementary figures and images for: Structural studies and molecular dynamics simulations suggest a processive mechanism of exolytic lytic transglycosylase from Campylobacter jejuni
Source: PLoS One. 2018 May 14;13(5):e0197136. doi: 10.1371/journal.pone.0197136 (PMC5951611; doi:10.1371/journal.pone.0197136)

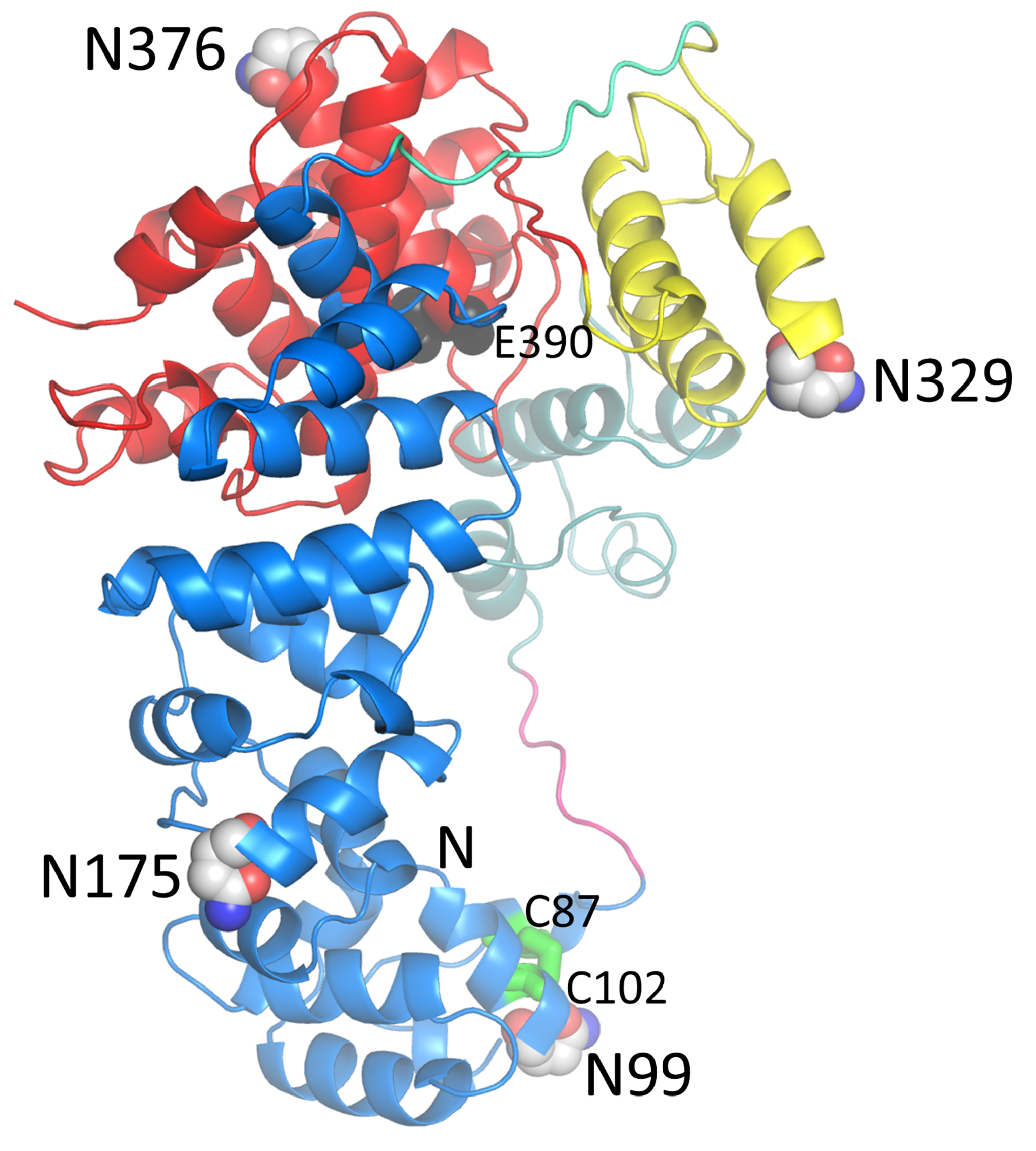

Supplement: S1 Fig — Native glycosylation sites N99, N175, N329, 376 are shown in spheres with grey carbon atoms. Catalytic E390 is shown in black spheres. The view is related to Fig 1B via a 90° rotation around the horizontal central axis. Domain colors are same as in Fig 1. (TIF) [file pone.0197136.s001.TIF]

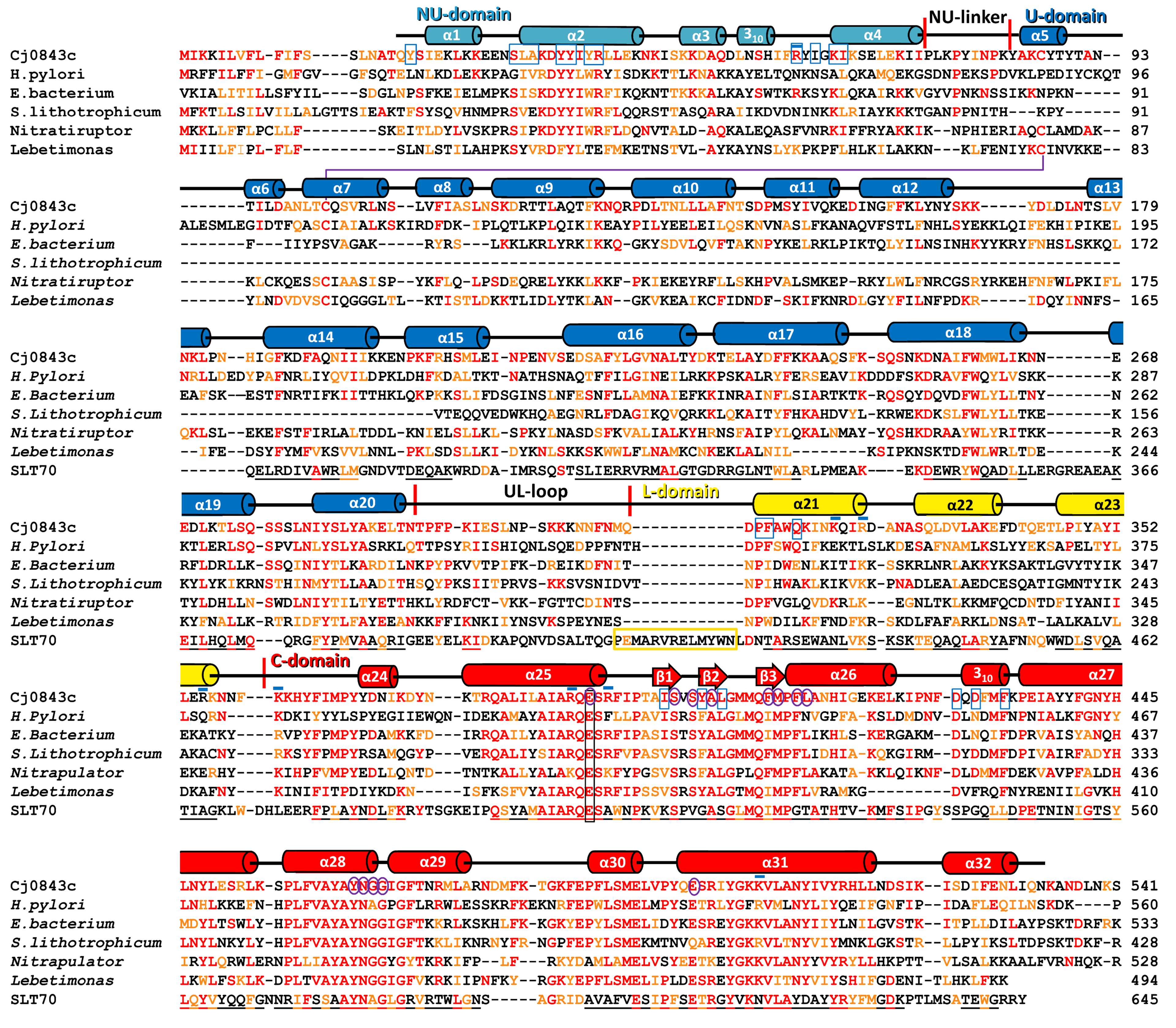

Supplement: S2 Fig — The alignment contains homologous LTs from Helicobacter pylori, Epsilonproteobacteria bacterium, Sulfurovum lithotrophicum, Nitratiruptor sp. SB155-2, Lebetimonas sp. JH292, and SLT70 from E. coli. The underlined SLT70 residues are in structurally equivalent positions in Cj0843. Residues are color-coded based on conservation; red residues are identical to the Cj0843, residues in orange are similar. The yellow boxed residues show the additional L-domain helix in SLT70. The C87-C102 disulfide bond is indicated in purple line. Blue boxes highlight residues at the interface between the NU-domain and the rest of Cj0843. Residues interacting either directly or indirectly (water-mediated) with bulgecin A are highlighted with a black oval. The black box shows the conserved catalytic glutamic acid. Positively charged residues with a blue bar above them are situated in the positively charged pocket 2. (TIF) [file pone.0197136.s002.TIF]

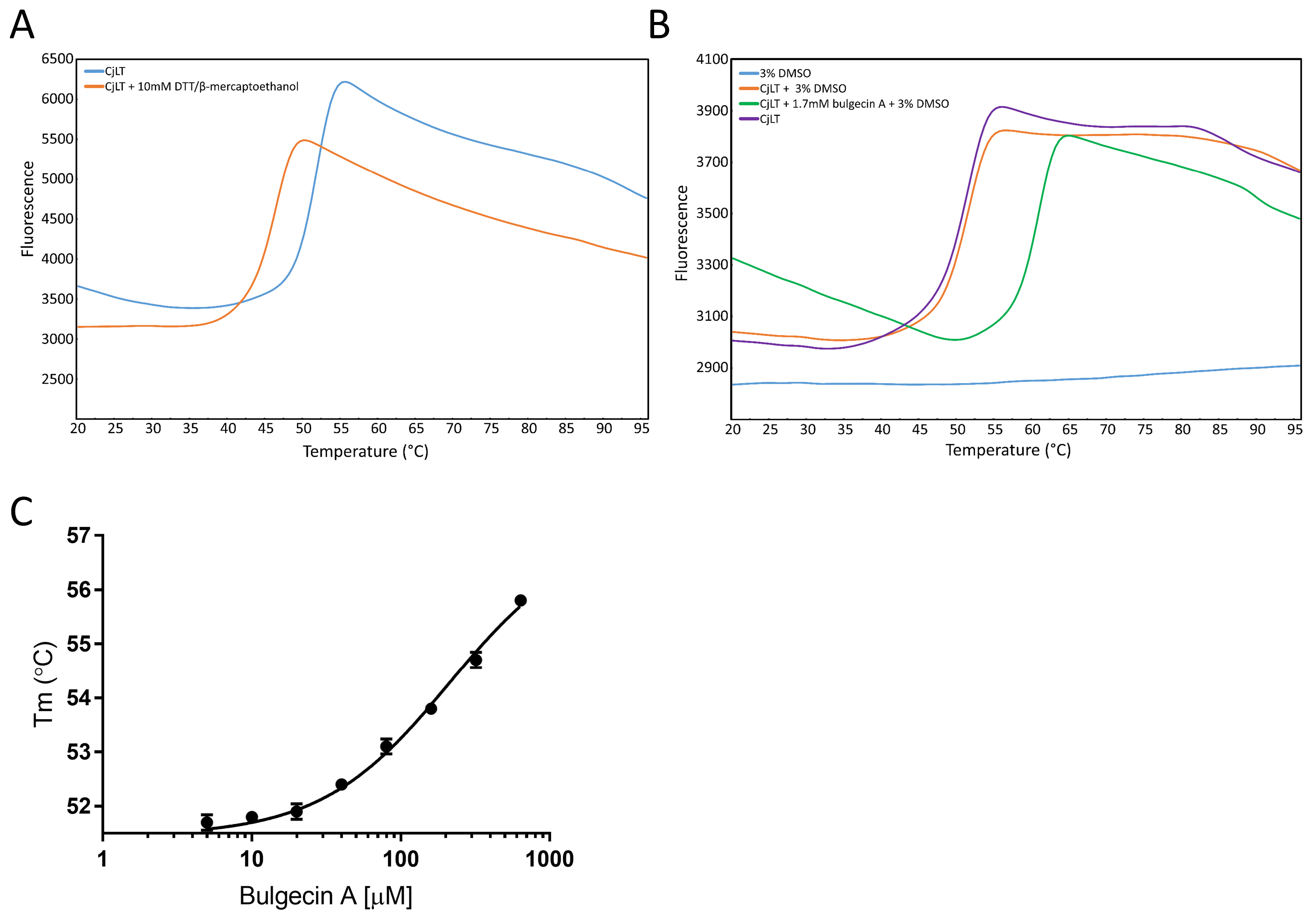

Supplement: S3 Fig — (A) DSF measurements of Cj0843 in the absence or presence of 10mM DTT and 10mM β-mercaptoethanol. (B) DSF measurements of Cj0843 in the absence or presence of 1.7mM bulgecin A; a 3% DMSO control is included. (C) DSF measurements of Cj0843 with varying concentrations of bulgecin A. Experiments were carried out in duplicate (1.67% DMSO included in the assays). (TIF) [file pone.0197136.s003.TIF]

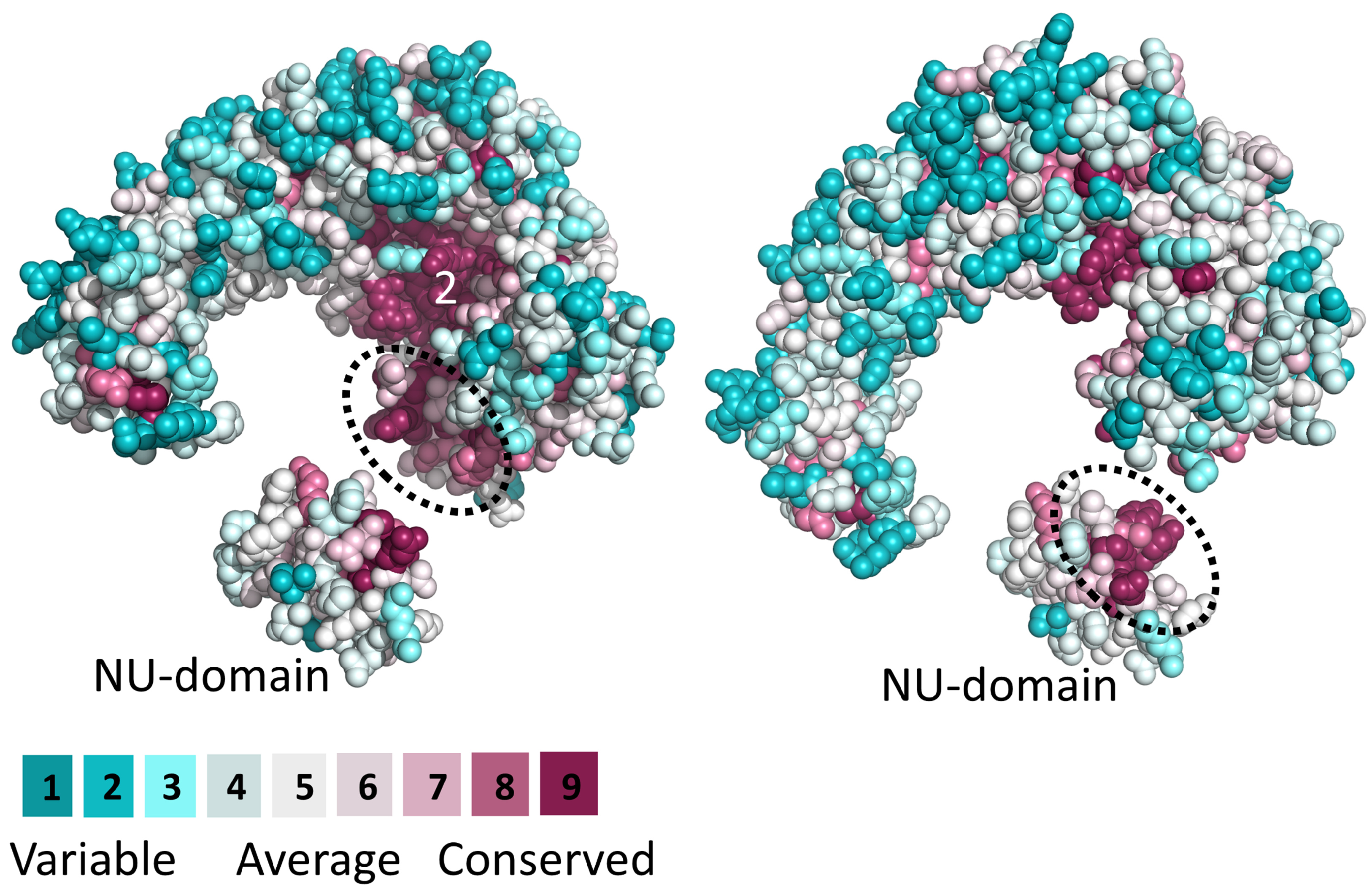

Supplement: S4 Fig — The degree of sequence conservation in Epsilonproteobacteria is color-coded onto the surface of the protein. The NU-domain, with the NU-loop removed, was moved 15Å away to expose the residues at the interface of C-domain, L-domain, and NU-domain. The figure was generated using ConSurf, and residue conservation ranges from non-conserved (dark blue/cyan) to conserved (dark magenta). The left and right figure are different orientations such that their views expose both sides of the C-/L-domain–NU-domain interface each highlighted by a dotted oval. Also conserved is the active site region labeled ‘2’ encompassing pocket 2 and the active site groove. (TIF) [file pone.0197136.s004.TIF]

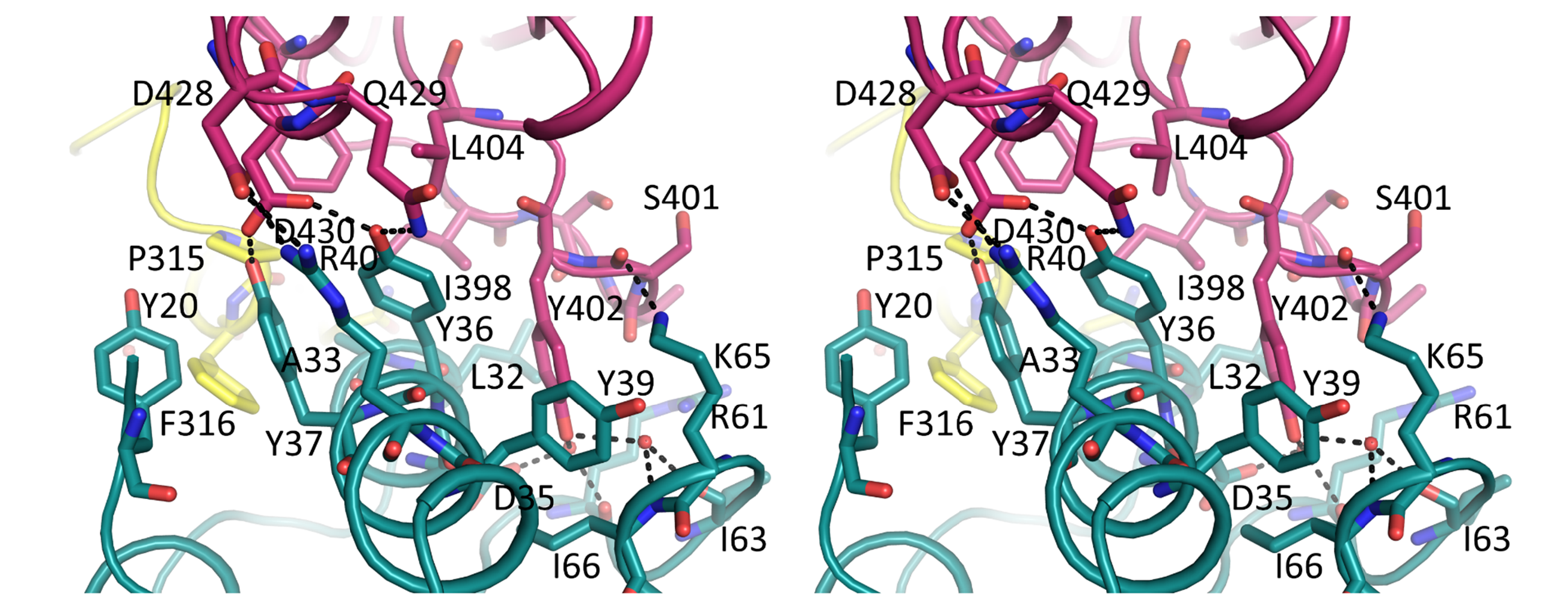

Supplement: S5 Fig — Color coding of the individual domains is as in Fig 1. (TIF) [file pone.0197136.s005.TIF]

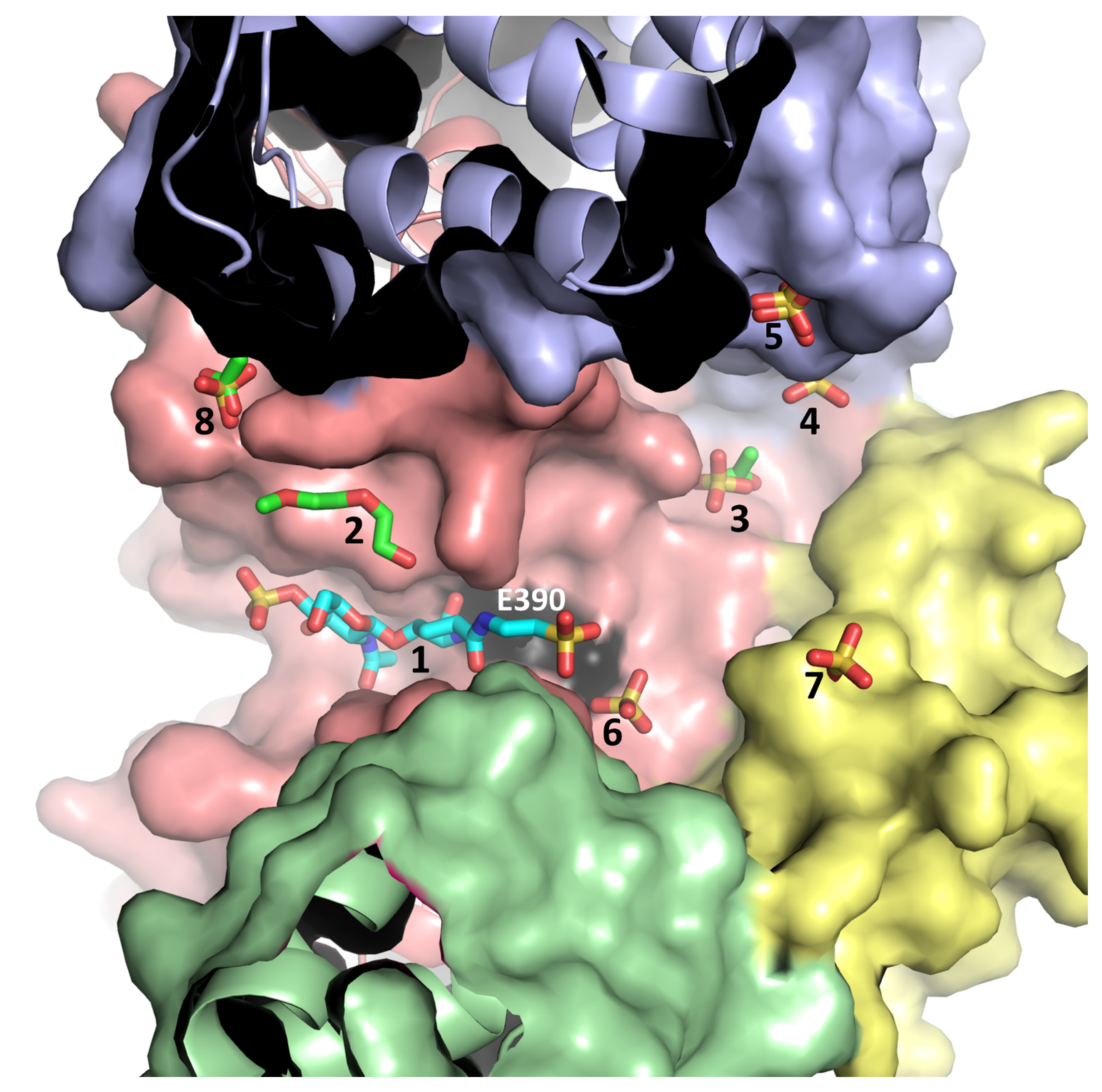

Supplement: S6 Fig — The following molecules were observed with their locations labeled in parentheses: Bulgecin A (1 with carbon atoms in cyan), a PEG molecule (2) and acetate ions (3 and 8; with carbon atoms in green) from the I23 space group structure, and sulfate ions (3–8) from either the bulgecin A complex or P3121 apo Cj0843 structure. Sites 3 to 7 are located in the positively charged pocket 2. The slabbed view into the active site is along an axis perpendicular to the axis of the doughnut-shaped Cj0843 similar to the view in Fig 3. The C-domain (red), L-domain (yellow), U-domain (blue), and NU-domain (blue/green) are shown with the surface patch comprised of atoms of E390 shown in black. (TIF) [file pone.0197136.s006.TIF]

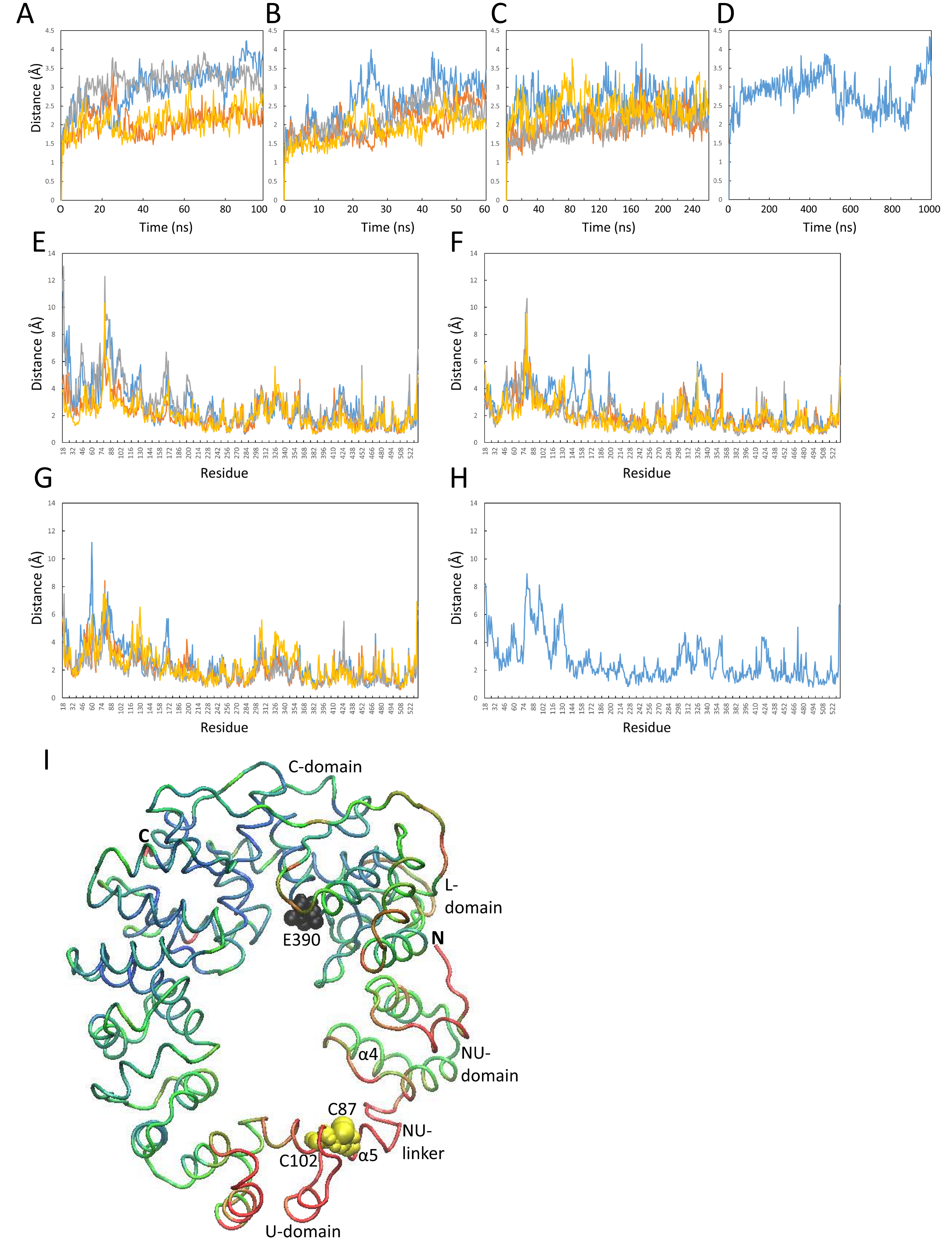

Supplement: S7 Fig — (A) 5 Disaccharide unit PG strand in the substrate-binding mode with deprotonated E390. (B) Same as A but with protonated E390. (C) 4 Disaccharide unit PG strand in the product-binding mode. (D) 1μs MD simulation of 5 disaccharide PG strand with an unbiased starting position. (E) RMSF per residue of the substrate MD runs with deprotonated E390. (F) Same as E but with protonated E390. (G) RMSF per residue of the product MD runs. (H) RMSF per residue of the 1μs MD simulation of 5 disaccharide PG strand in an unbiased starting position. The runs 1, 2, 3, and 4 of the substrate and product MD simulations in A-C and E-G are colored blue, orange, grey, and yellow, respectively. (I) Tube representation of Cj0843 at the end of the 1μs MD simulation color-coded by average RMSF per residue ranging from 0.5 to 5.0Å corresponding to blue-green-red, respectively. The Cj0843 orientation is similar as in Fig 1A. (TIF) [file pone.0197136.s007.TIF]

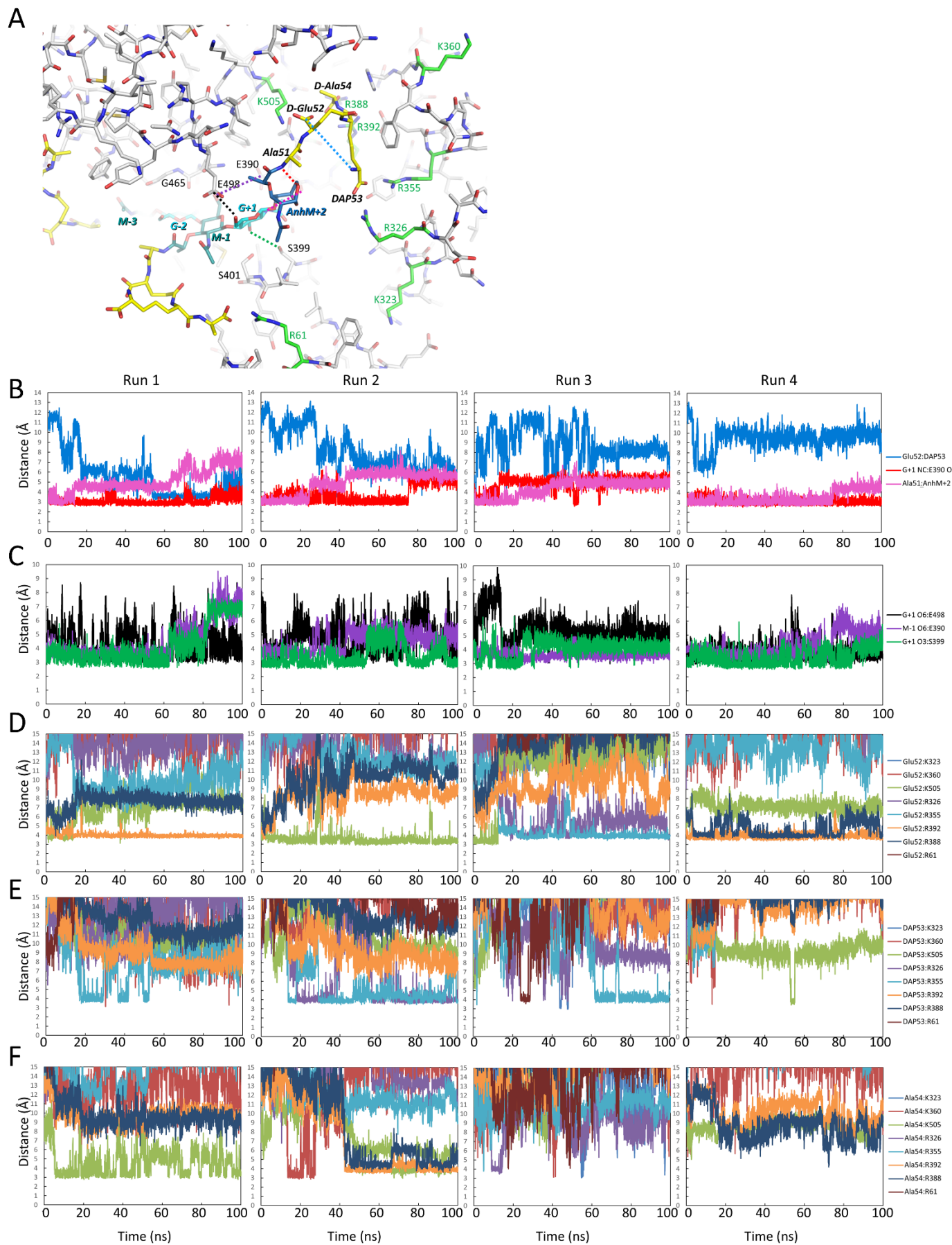

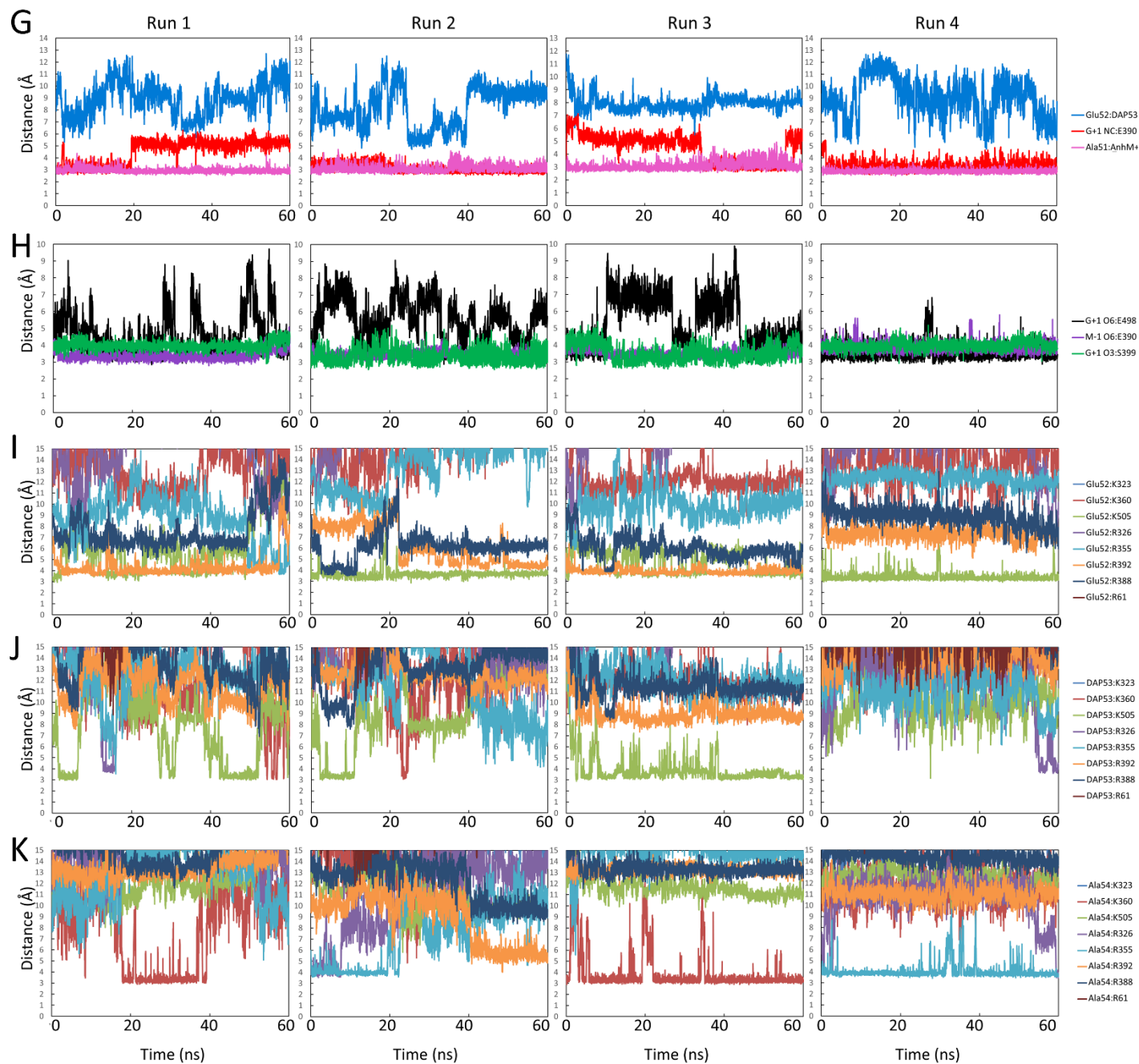

Supplement: S8 Fig — The catalytic E390 was either deprotonated (B-F) or protonated (G-K). (A) Close-up view of pocket 2 area of the active site of Cj0843 with PG strand in substrate-binding mode. Color coding and orientation are the same as in Fig 6A except a more zoomed in view is shown. Distances that are monitored are shown as colored dashed lines and correspond to those in subsequent panels B, C, G, and H. (B) Distances plotted versus time between the CD atom of PG Glu52 and NZ atom of PG DAP53 (blue), the N-acetyl nitrogen atom of GlcNAc+1 and the backbone O atom of E390 (red), the nitrogen atom of PG Ala51 and the O6 atom of AnhMurNAc+2 (magenta). (C) Distances are shown for the O6 atom of GlcNAc+1 and the CD atom of E498 (black), O6 atom of MurNAc-1 and the CD atom of E390 (magenta), and the O3 atom of GlcNAc+1 and the OH atom of S399 (green). (D) Distances of the carboxyl CD of PG Glu52 and the NZ atoms of indicated K residues or CZ atoms of indicated R residues. (E) Same as (D) but with PG carboxyl atom CZ of DAP53. (F) Same as (D) but with PG carboxyl C atom of Ala54. (G)-(K) Same as (B)-(F) but with protonated E390 residue during the simulations. (PDF) [file pone.0197136.s008.pdf]

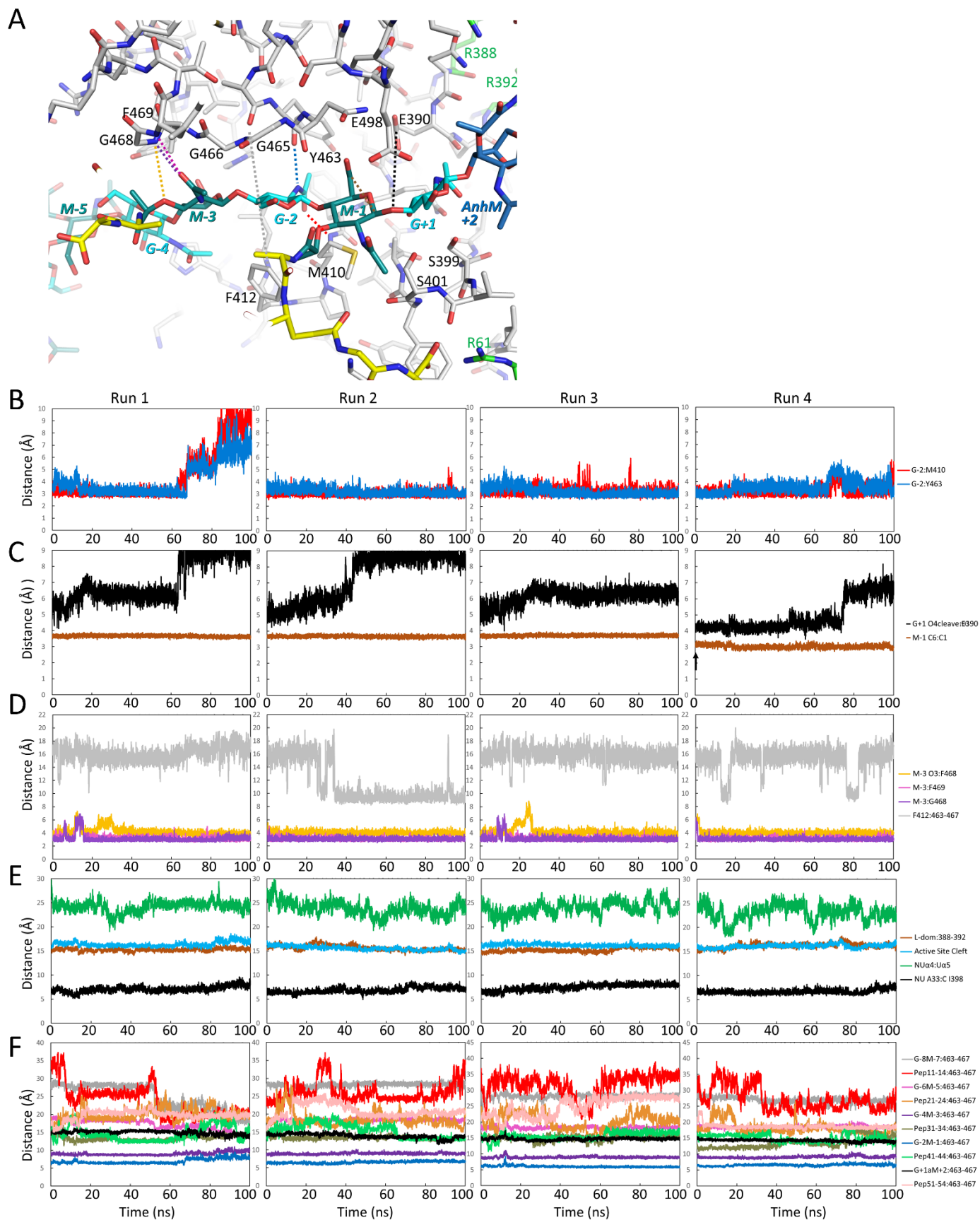

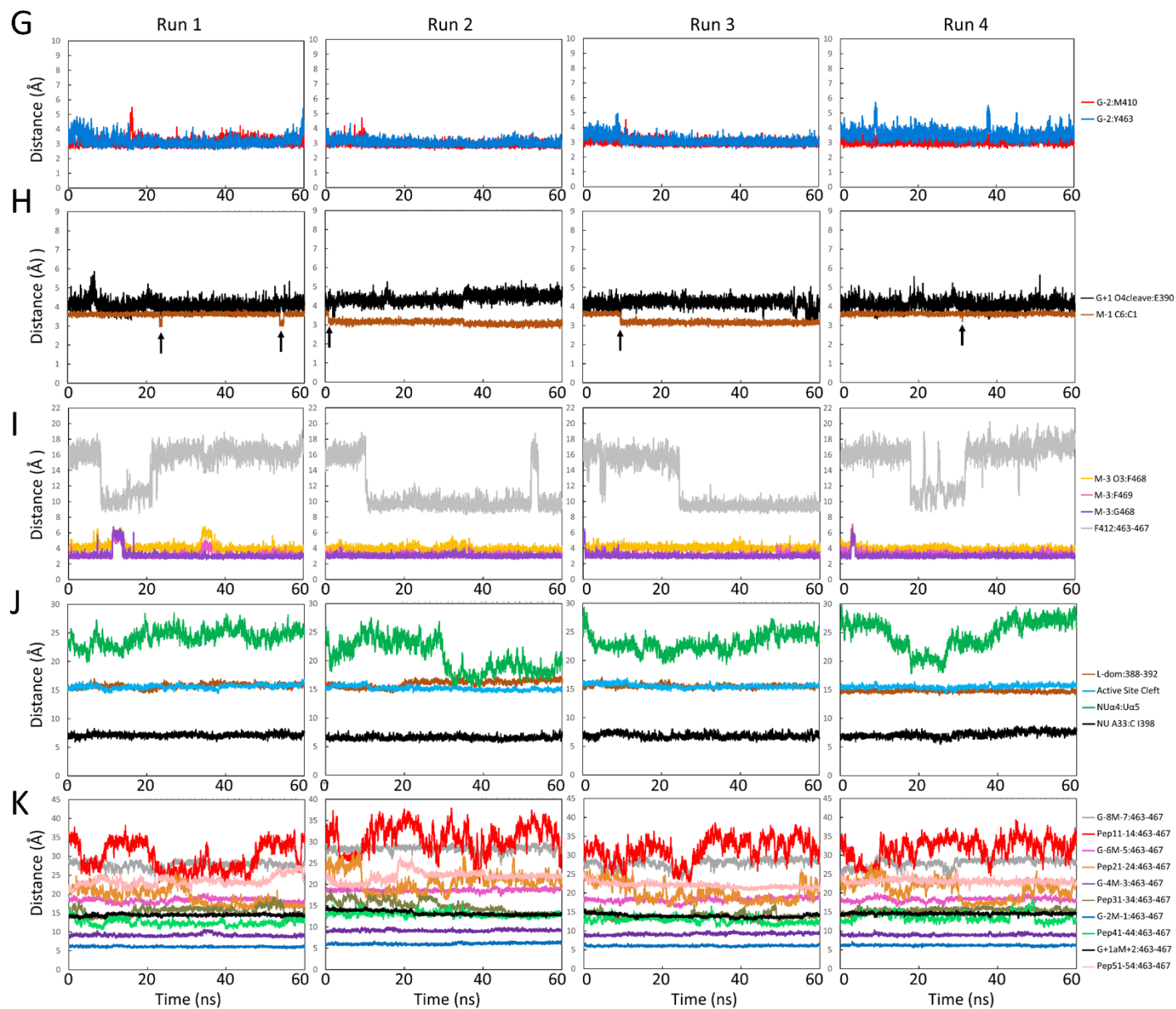

Supplement: S9 Fig — The catalytic E390 was either deprotonated (B-F) or protonated (G-K). (A) Close-up view of active site groove of Cj0843 with PG strand in substrate-binding mode. Distances that are monitored are shown as colored dashed lines and correspond to those in subsequent panels B-D and G-I. (B) Distances plotted versus time between the oxygen of the N-acetyl moiety of GlcNAc-2 and the backbone nitrogen of M410 (red) and between the nitrogen of the N-acetyl moiety of GlcNAc-2 and the backbone oxygen of Y463 (blue). (C) Distances of the O4 atom of GlcNAc+1, belonging to the bond to be cleaved, and the CD atom of E390 (black), and between the C6 and C1 atoms of MurNAc-1 indicative of a chair or boat conformation (brown). A black arrow indicates instances where a boat conformation starts. (D) Distances between the O3 atom of MurNAc-3 and the backbone nitrogen of F468 (yellow), the oxygen atom of the N-acetyl moiety of MurNAc-3 and the backbone nitrogen of F469 (pink), the oxygen atom of the N-acetyl moiety of MurNAc-3 and the backbone nitrogen of G468 (magenta), and the CZ atom of F412 and the center of mass of residues 463–467 (grey). (E) Distances between the center of mass of the L-domain and the center of mass of residues 388–392 (brown), the center of mass of residues 398–403 & 409–413 with respect to the center of mass of residues 460–470 & 497–502 representing the width of the active site clef (light blue), center of mass of NU-domain helix α4 and center of mass U-domain helix α5 representing the distance across the flexible NU-loop (green), and the distance of the CA atom of NU-domain A33 and CA atom of C-domain I398 representing the distance across the NU-domain interface. (F) Distances of the 5 PG disaccharide units (GxMy) and their respective tetrapeptide moieties (Pepxx-yy) with respect to the center of mass of active site residues 463–467. (G)-(K) Same as (B)-(F) but with protonated E390 during MD runs. (PDF) [file pone.0197136.s009.pdf]

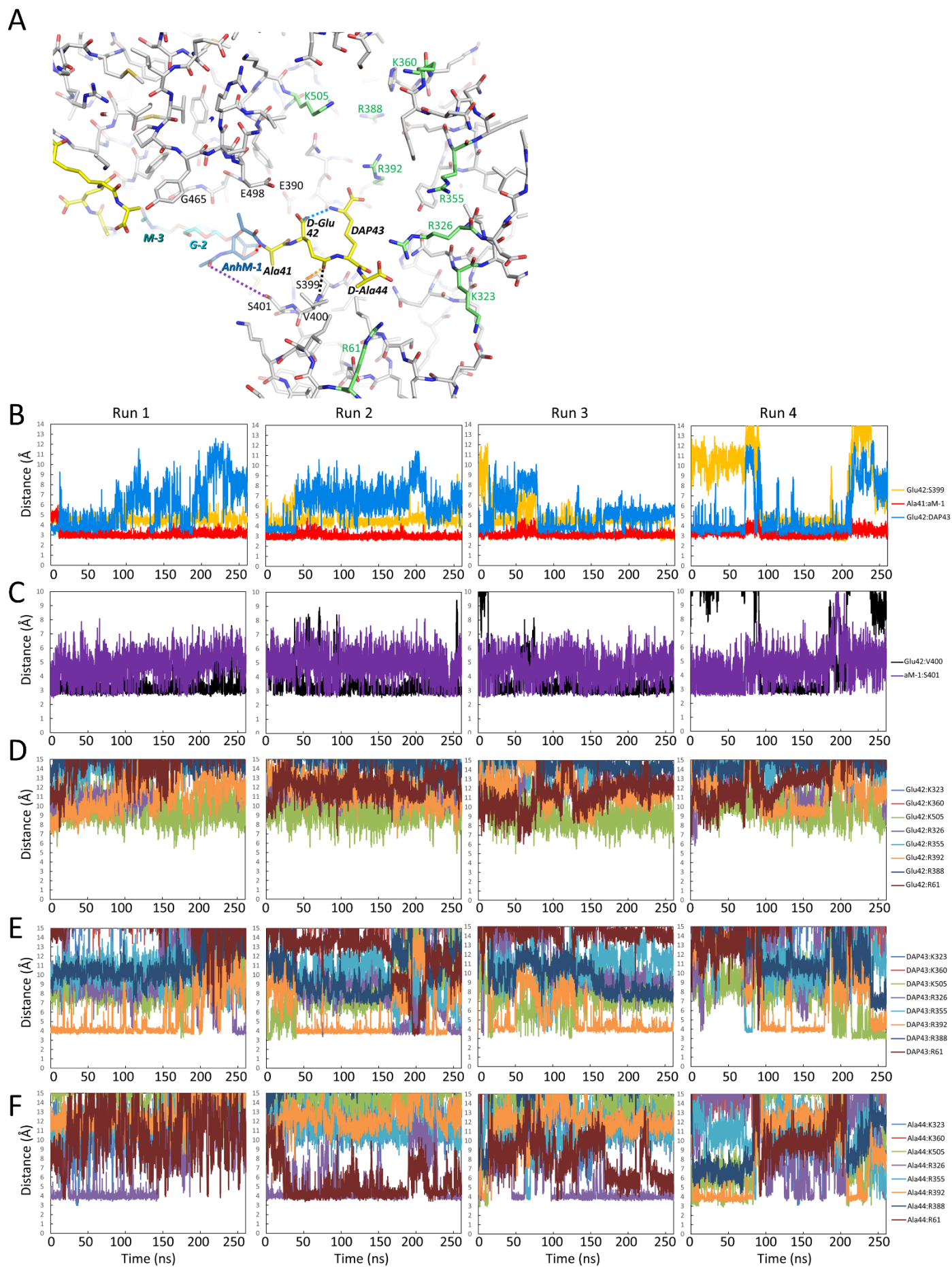

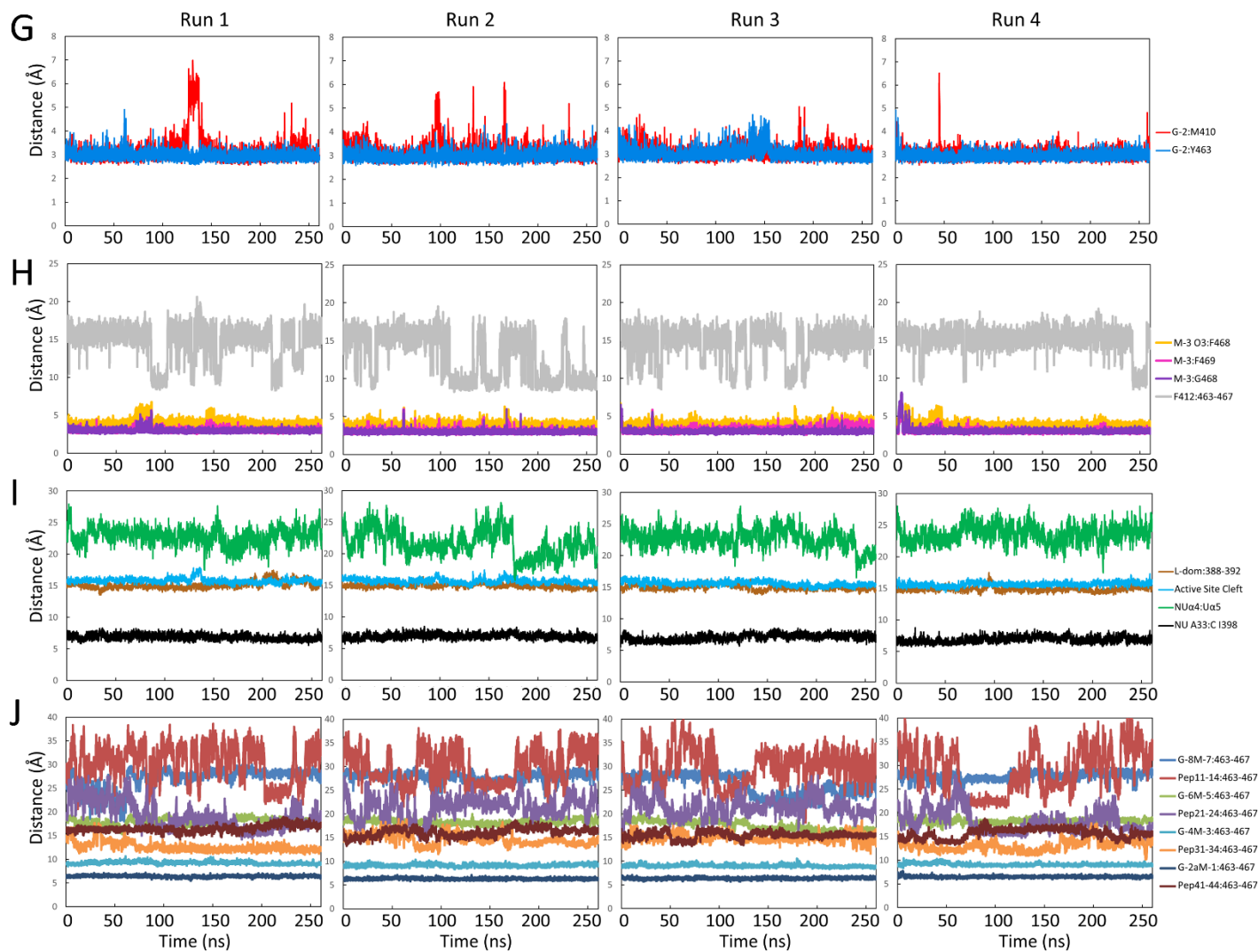

Supplement: S10 Fig — (A) Close-up view of pocket 2 area of the active site of Cj0843 with PG strand in product-binding mode. Distances that are monitored are shown as colored dashed lines. The view and atom coloring are as in Fig 6B but a more zoomed in view is shown. (B) Distances plotted versus time between the oxygen atom of PG Glu42 and the OH group of S399 (orange), the nitrogen atom of PG Ala41 and the O6 atom of AnhMurNAc-1 (red), and the CD atom of PG Glu42 and NZ atom of PG DAP43 (blue). (C) Distances between the oxygen atom of PG Glu42 and the backbone nitrogen of V400 group of S399 (black), and the oxygen atom of the N-acetyl moiety of AnhMurNAc-1 and the OH group of S401 (magenta). (D)-(F) Same as in Figures D-F in S8 Fig but now with the PG carboxylates from tetrapeptide residues 42–44, respectively. (G) Same as in Figure B in S9 Fig. (H)-(I) Same as in Figures D-E in S9 Fig. (J) Same as Figure F in S9 Fig but now lacking the cleaved off terminal disaccharide PG unit. (PDF) [file pone.0197136.s010.pdf]

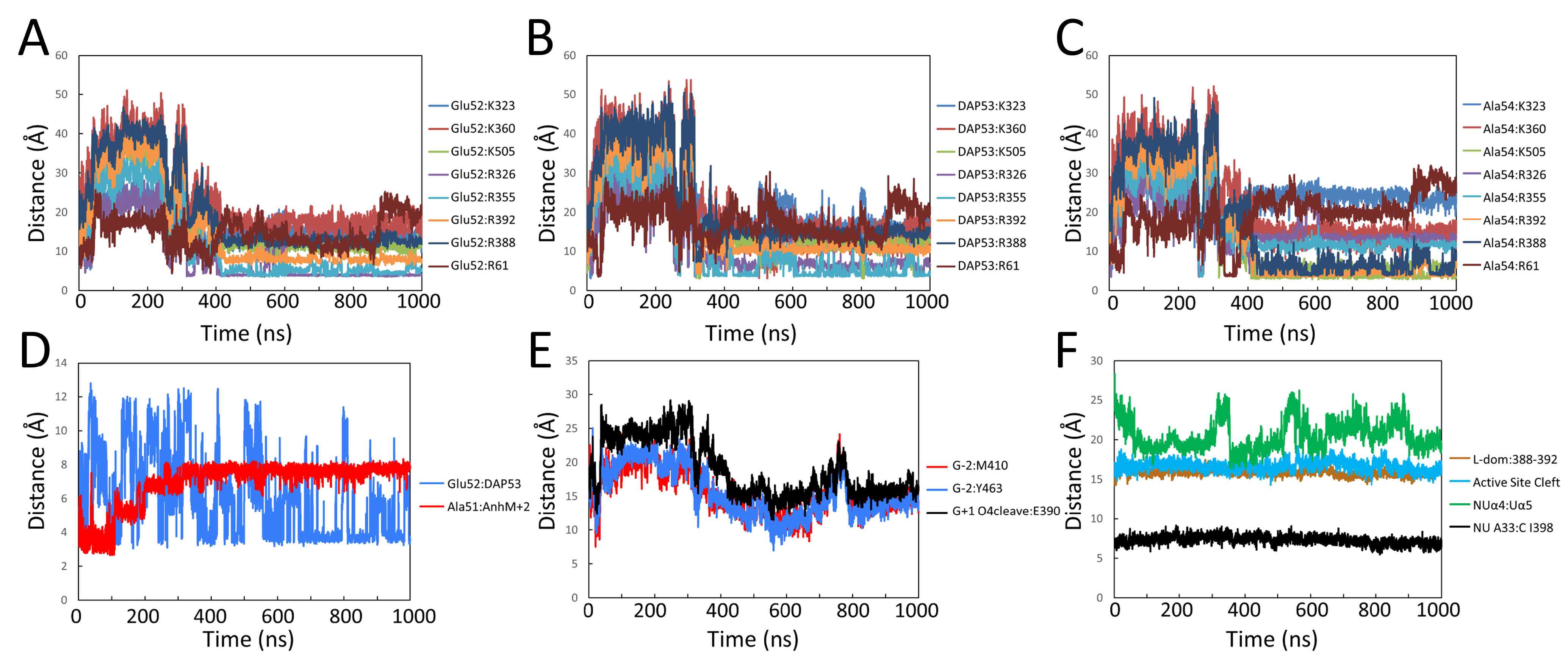

Supplement: S11 Fig — Distances plotted versus time. (A)-(C) Same as Figures D-F in S8 Fig. (D) Distances plotted versus time between the CD atom of PG Glu52 and NZ atom of PG DAP53 (blue), and the nitrogen atom of PG Ala51 and the O6 atom of AnhMurNAc+2 (red). (E) Distances between the oxygen of the N-acetyl moiety of GlcNAc-2 and the backbone nitrogen of M410 (red), the nitrogen of the N-acetyl moiety of GlcNAc-2 and the backbone oxygen of Y463 (blue), and distances of the O4 atom of GlcNAc+1, belonging to the bond to be cleaved, and the CD atom of E390 (black). (F) Same as in Figure E and S9 Fig. (TIF) [file pone.0197136.s011.TIF]

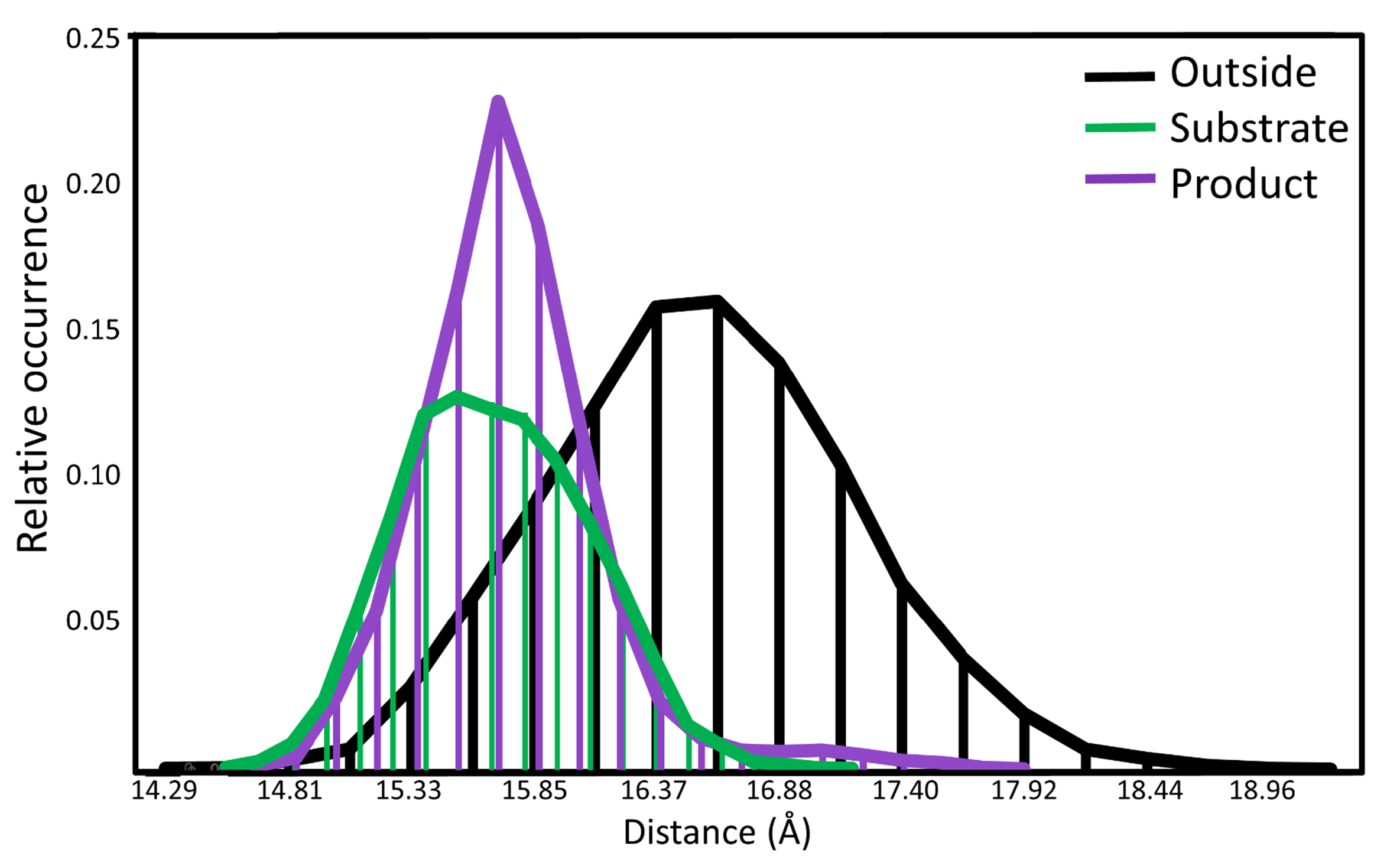

Supplement: S12 Fig — The width of the active site is defined as the center of mass of residues 398–403 & 409–413 relative to the center of mass of residues 460–470 & 497–502. Twenty distance bins were plotted for the Cj0843 active site width as observed in the substrate-binding mode simulation (green), product-binding mode simulation (magenta), and the 1μs simulation in which the starting PG position was placed outside the central hole of Cj0843 (black). (TIF) [file pone.0197136.s012.TIF]
